# The vibronic state dependent predissociation of H_2_S: determination of all fragmentation processes†

**DOI:** 10.1039/d2sc06988a

**Published:** 2023-02-14

**Authors:** Yarui Zhao, Junjie Chen, Zijie Luo, Yao Chang, Jiayue Yang, Weiqing Zhang, Guorong Wu, Stuart W. Crane, Christopher S. Hansen, Hongbin Ding, Feng An, Xixi Hu, Daiqian Xie, Michael N. R. Ashfold, Kaijun Yuan, Xueming Yang

**Affiliations:** a School of Physics, Key Laboratory of Materials Modification by Laser, Ion and Electron Beams, Chinese Ministry of Education, Dalian University of Technology Dalian 116024 China; b State Key Laboratory of Molecular Reaction Dynamics and Dalian Coherent Light Source, Dalian Institute of Chemical Physics, Chinese Academy of Sciences Dalian 116023 China kjyuan@dicp.ac.cn; c Institute of Theoretical and Computational Chemistry, Key Laboratory of Mesoscopic Chemistry, School of Chemistry and Chemical Engineering, Nanjing University Nanjing 210023 China; d School of Chemistry, University of Bristol Bristol BS8 1TS UK mike.ashfold@bristol.ac.uk; e School of Chemistry, University of New South Wales Sydney NSW 2052 Australia; f Kuang Yaming Honors School, Institute for Brain Sciences, Jiangsu Key Laboratory of Vehicle Emissions Control, Center of Modern Analysis, Nanjing University Nanjing 210023 China xxhu@nju.edu.cn; g Hefei National Laboratory Hefei 230088 China; h Department of Chemistry, Southern University of Science and Technology Shenzhen 518055 China

## Abstract

Photochemistry plays a significant role in shaping the chemical reaction network in the solar nebula and interstellar clouds. However, even in a simple triatomic molecule photodissociation, determination of all fragmentation processes is yet to be achieved. In this work, we present a comprehensive study of the photochemistry of H_2_S, derived from cutting-edge translational spectroscopy measurements of the H, S(^1^D) and S(^1^S) atom products formed by photolysis at wavelengths across the range 155–120 nm. The results provide detailed insights into the energy disposal in the SH(*X*), SH(*A*) and H_2_ co-fragments, and the atomisation routes leading to two H atoms along with S(^3^P) and S(^1^D) atoms. Theoretical calculations allow the dynamics of all fragmentation processes, especially the bimodal internal energy distributions in the diatomic products, to be rationalised in terms of non-adiabatic transitions between potential energy surfaces of both ^1^A′ and ^1^A′′ symmetry. The comprehensive picture of the wavelength-dependent (or vibronic state-dependent) photofragmentation behaviour of H_2_S will serve as a text-book example illustrating the importance of non-Born–Oppenheimer effects in molecular photochemistry, and the findings should be incorporated in future astrochemical modelling.

## Abstract 2

The comprehensive picture of the fragmentation behaviour of H_2_S has been provided by detecting the H, S(^1^D) and S(^1^S) atom products at wavelengths 155–120 nm.

## Graphical abstract

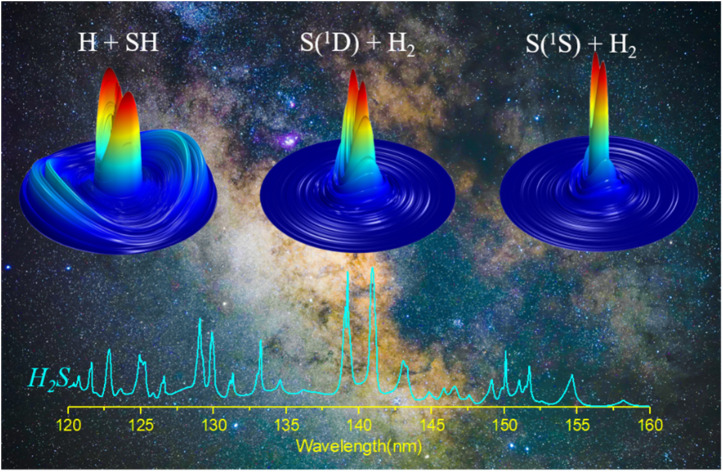

## Introduction

Sulfur is one of the most abundant elements in the universe and its presence in the interstellar medium (ISM) has been widely studied.^1,2^ Estimates based on the limited range of S-containing compounds detected in low-density diffuse clouds imply sulfur fractions similar to the cosmic value.^3^ However, the number densities of S-containing molecules in denser regions of the ISM imply much lower fractional abundances.^4–7^ For example, the estimated abundances of S-containing species in the outer layers of the photodissociation region in the Horsehead nebula are only ∼25% of the cosmic value^8^ and orders of magnitude lower abundances have been suggested in cold molecular clouds.^9–11^ This apparent depletion is explained by assuming that much of the (undetected) sulfur is locked up in solids, *i.e.* in dust grains and in icy mantles. The high abundance and mobility of hydrogen in an ice matrix leads to the expectation that most of the sulfur released from interstellar ice mantles (by sputtering, thermal and/or photo-induced desorption) will be in the form of H_2_S.^12–15^ Consistent with such expectations, H_2_S has been detected in the atmospheres of comets (like P/Halley,^16^ C/1995 O1 (Hale–Bopp),^17,18^ C/2014 Q2 (Lovejoy)^19^ and 67P/Churyumov–Gerasimenko^20^) and planets (*e.g.* Jovian,^21^ Uranus,^22^ and Neptune^23^).

The local S atom and SH radical abundances in the ISM are strongly linked with the photodissociation of H_2_S by solar vacuum ultraviolet (VUV) photons. Current astrochemical models^24^ based on the limited prior knowledge propose that photon absorption by H_2_S causes dissociation to, exclusively, H + SH fragments at all energies up to the first ionization potential (84 432 ± 2 cm^−1^ (ref. 25)). However, our recent photofragment translational spectroscopy (PTS) studies^26^ revealed the over-simplicity of the current model descriptions. The PTS experiments confirmed S–H bond fission as the dominant photodissociation process, but also revealed, for example, the rich quantum state dependent photofragmentation dynamics of H_2_S that can prevail even when exciting within just one predissociated electronic state.^27^ Any revision of astrochemical models requires a full understanding of the fragmentation dynamics of H_2_S across the whole solar VUV region. Such an overall picture can now be achieved using the VUV free electron laser (FEL) at the Dalian Coherent Light Source (DCLS).^28^

**Fig. 1:**
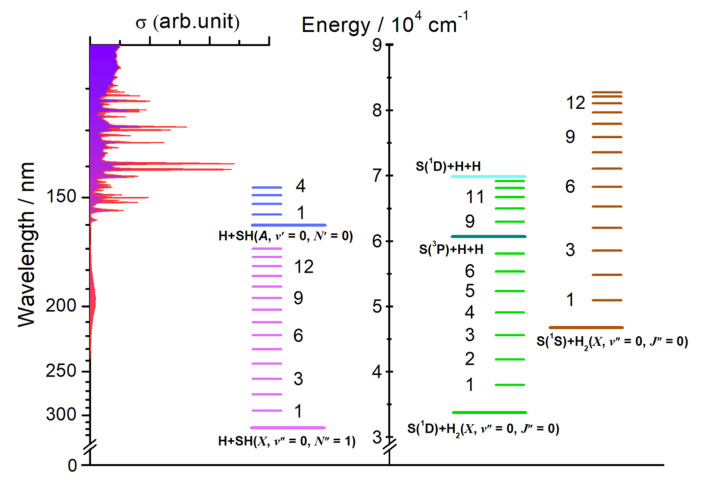
H_2_S absorption cross-section *versus* wavelength (left, after ref. 44), together with (right) the energetic thresholds for each of dissociation processes (1)–(6) and for forming the H_2_ and SH products in different vibrational states.

H_2_S and H_2_O are the two lightest group 16 (VIA) hydrides. The wavelength-dependent photofragmentation dynamics of H_2_O molecules have been studied extensively, both experimentally and theoretically, to the extent that this system now provides several textbook illustrations of the importance of non-adiabatic effects (conical intersections (CIs) between potential energy surfaces (PESs)) in determining molecular photochemistry.^29–42^ In contrast, photodissociation dynamics of H_2_S are limited. The UV absorption spectrum of H_2_S shows many similarities to that of H_2_O, but some important differences have already been recognised. All the characteristic features – the two continua and the onset of sharp Rydberg resonances – are shifted to longer wavelengths. The cross-section of the long wavelength continuum maximizes at *λ* ∼ 195 nm and thereafter declines to shorter wavelengths (Fig. 1).^43–45^ Prior photodissociation studies at wavelengths *λ* ≥ 198 nm revealed prompt S–H bond fission and formation of ground (*X*^2^Π) state SH radicals.^46^ The SH(*X*) radicals are formed mostly in their lowest (*v*′′ = 0) vibrational level with little rotational excitation. This energy disposal reflects one key difference in the electronic structure of H_2_O and H_2_S. Vertical excitation from the 2b_1_ HOMO of H_2_S at the ground state equilibrium geometry samples not one but two states that are near degenerate in the Franck Condon region (with ^1^B_1_ and ^1^A_2_ symmetry in *C*_2v_, *i.e.* both ^1^A′′ in *C*_s_), only one of which is dissociative upon H–SH bond extension.^47^ PTS studies following excitation at *λ* = 157.6 nm also revealed prompt S–H bond fission and formation of H + SH(*X*) radicals.^48^ These SH(*X*) radicals are formed both vibrationally and rotationally excited, however, with the SH(*X*, *v*′′ = 0 and 1) products showing clearly bimodal rotational state population distributions which have been rationalised by invoking two different formation mechanisms. PTS studies following excitation at *λ* = 121.6 nm, in contrast, revealed no SH(*X*) products but formation of electronically excited SH(*A*^2^Σ^+^) radicals, with broad rotational state population distributions extending up to ‘super rotor’ levels above the dissociation energy of the SH(*A*) state – *i.e.* rotational levels that exist only by virtue of the accompanying centrifugal potential energy barrier.^49,50^ Though rich information of H_2_S photodissociation has been acquired, a comprehensive picture for all fragmentation channels is yet to be established.

Here we employ three different PTS methods to monitor the atomic products formed upon photolysis of jet-cooled H_2_S molecules at many different wavelengths in the range 154.5 ≥ *λ* ≥ 121.6 nm. The experimental procedures have all been described previously and are thus confined to the ESI.† The results offer a ‘big picture’ view of the fragmentation dynamics, as functions of excitation energy and parent vibronic level. We deduce that excitation of the first continuum (1^1^A′′/2^1^A′′) directly, or indirectly following non-adiabatic coupling from a photoexcited Rydberg state (R), results in H + SH(*X*) products. H + SH(*A*) products arise from molecules that dissociate on a higher valence (2^1^A′) PES of H_2_S. ‘Orbiting’-type motions of an emerging H atom about its SH partner on the 2^1^A′ PES can funnel dissociating molecules towards linear configurations (H⋯SH and H⋯HS geometries) and thereby promote coupling to the 1^1^A′′ PES *via* a (Renner–Teller) seam of degeneracy or to the *X̃* state PES *via* the CIs at extended H–SH and H–HS bond lengths, and eventual formation of highly rovibrationally excited SH(*X*) and H_2_ products. The topography of the 2^1^A′ PES also supports a rival route to S(^1^D) products, wherein symmetric S–H bond extension in tandem with a reduction in the interbond angle leads to the elimination of highly vibrationally excited H_2_ fragments. The present work provides the most detailed and complete picture of the photofragmentation behaviour of H_2_S molecules yet available, the results of which should be incorporated in future astrochemical modelling.

## Results and discussion

Eqn (1)–(6) list all possible spin-allowed fragmentation processes of H_2_S in the photolysis wavelength range studied in this work,

1 H_2_S → H + SH(*X*^2^Π_3/2_, *v*′′ = 0, *N*′′ = 1), (*E*_th_ = 31451 ± 4 cm^−1^)

2 → H + SH(*A*^2^Σ, *v*′ = 0, *N*′ = 0), (*E*_th_ = 62284 ± 4 cm^−1^)

3 → H + H + S(^3^P_2_), (*E*_th_ = 60696 ± 25 cm^−1^)

4 → H + H + S(^1^D_2_), (*E*_th_ = 69935 ± 25 cm^−1^)

5 → H_2_(*X*^1^Σ_g_^+^, *v*′′ = 0, *J*′′ = 0) + S(^1^D_2_), (*E*_th_ = 33817 ± 25 cm^−1^)

6 → H_2_(*X*^1^Σ_g_^+^, *v*′′ = 0, *J*′′ = 0) + S(^1^S_0_), (*E*_th_ = 46758 ± 25 cm^−1^)

The threshold energies (*E*_th_) for these fragmentation channels are given in parentheses.^26,27^ For orientation, these threshold energies are also included in Fig. 1, along with the energies of selected rovibrational levels of the various diatomic products. In what follows, we report data from three different experimental methods and from complementary high-level *ab initio* electronic structure calculations (summarised in the ESI†) that, together, offer a step change in understanding of the photofragmentation dynamics of this prototypical hydride molecule.

### (a) Time-of-flight measurements of H atom products

**Fig. 2:**
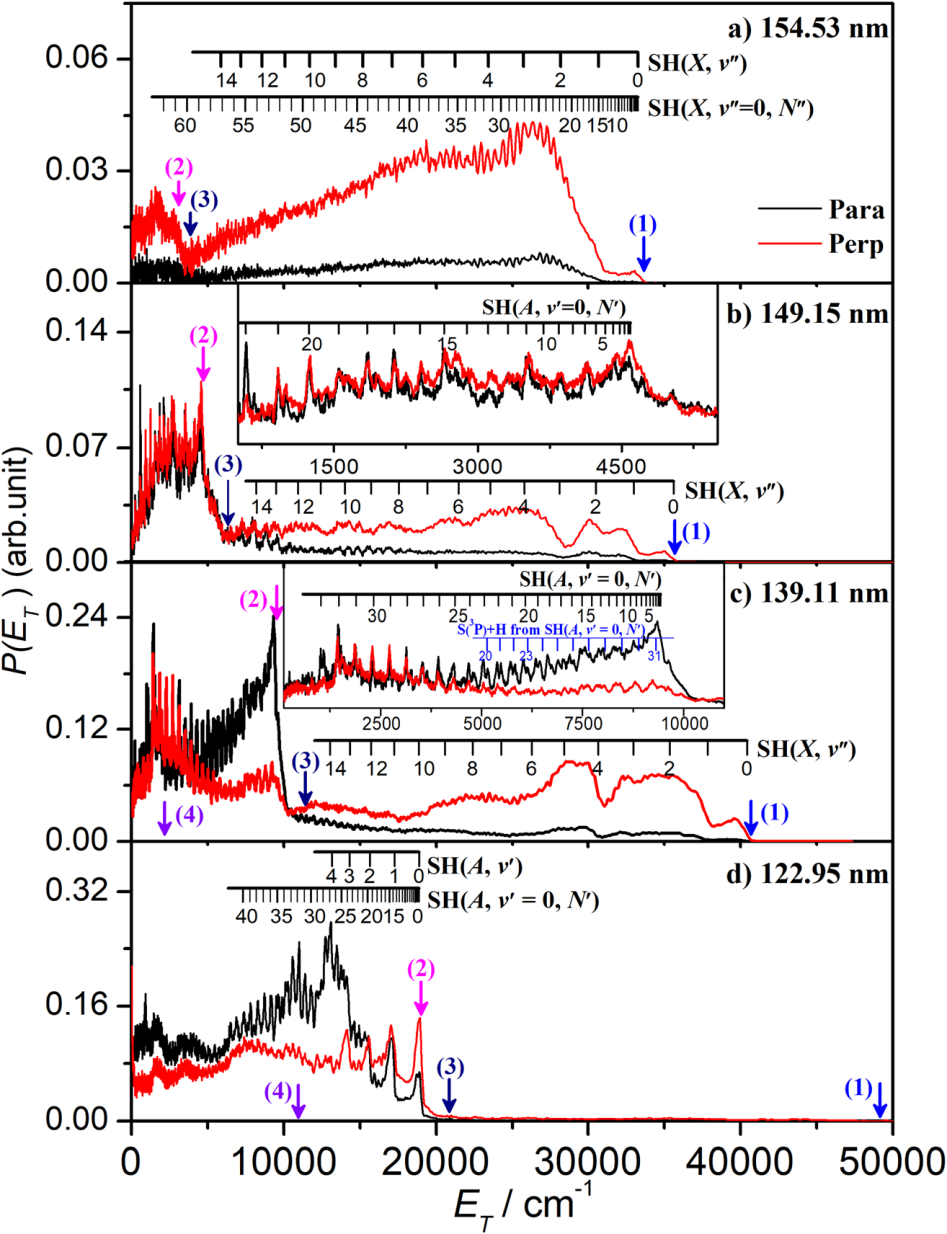
The *P*(*E*_T_) spectra following photodissociation of H_2_S at *λ* = (a) 154.53 nm, (b) 149.15 nm, (c) 139.11 nm and (d) 122.95 nm, with the detection axis aligned parallel (black) and perpendicular (red) to the polarisation (***ε***) vector of the photolysis laser radiation. The insets in (b) and (c) show expanded views of the low energy parts of the respective *P*(*E*_T_) spectra. The combs displayed above these spectra show the *E*_T_ values associated with formation of H atoms together with selected rovibrational levels of the primary SH(*X*) and SH(*A*) fragments and, in (c), with H atoms formed by predissociation of primary SH(*A*, *v*′ = 0, *N*′) fragments. The maximum *E*_T_ values associated with each of channels (1) – (4) are shown by vertical blue, red, navy and violet arrows.

H atom time-of-flight (TOF) spectra were recorded following excitation of H_2_S molecules at many wavelengths chosen to overlap with peaks in the absorption spectrum (Fig. 1, reproduced on an expanded scale in Fig. S1†). The TOF data were then converted into the H atom translational energy distributions, *P*(*E*_T_), where7
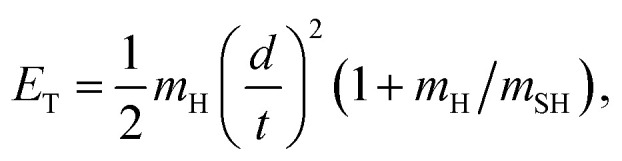
*m*_H_ and *m*_SH_ are the respective photofragment masses, *d* is the distance separating the interaction region and the detector, and *t* is the H atom TOF measured over this distance. Fig. 2 shows *P*(*E*_T_) spectra obtained at wavelengths *λ* = (a) 154.53 nm, (b) 149.15 nm, (c) 139.11 nm and (d) 122.95 nm, with the polarization (***ε***) vector of the photolysis radiation aligned both parallel (θ = 0°) and perpendicular (θ = 90°) to the detection axis [note, to avoid confusion, we use a non-italic θ to define the recoil velocity direction and, later, an italic *θ* to define the ∠HSH bond angle]. The internal energy of the primary SH fragments, *E*_int_, associated with any given *E*_T_ in these spectra is given by

8 *E*_int_ = *E*_phot_ − *E*_th_(1) − *E*_T_

where *E*_phot_ is the photolysis photon energy and *E*_th_(1) is the threshold energy for forming H + SH(*X*) products. The *P*(*E*_int_) distributions derived from the four *P*(*E*_T_) distributions shown in Fig. 2 and from other ten wavelengths in the range 154.53 ≥ *λ* ≥ 122.95 nm are all shown in Fig. S2–S4.† We now describe the main wavelength-dependent trends.

The energetic threshold for process (2) would correspond to an excitation wavelength *λ* ∼ 160.5 nm. However, the SH fragments formed at the longest wavelengths investigated in the present work (*e.g. λ* = 154.53 nm, Fig. 2(a)) are exclusively in their electronic ground state. These H + SH(*X*) fragments show a strong preference for recoiling along an axis perpendicular to ***ε***. The combs festooned above the spectra in Fig. 2(a) show the SH(*X*) population distributed over a very wide range of vibrational (*v*′′) and rotational (*N*′′) quantum states. The SH(*X*) potential energy curve correlates with H + S(^3^P) fragments at infinite separation and the small feature at low *E*_T_ in Fig. 2(a) is consistent with the three-body dissociation to 2H + S(^3^P) products, the threshold energy for which (*E*_th_(3)) lies ∼1600 cm^−1^ below that for two-body dissociation to H + SH(*A*) products.

**Fig. 3:**
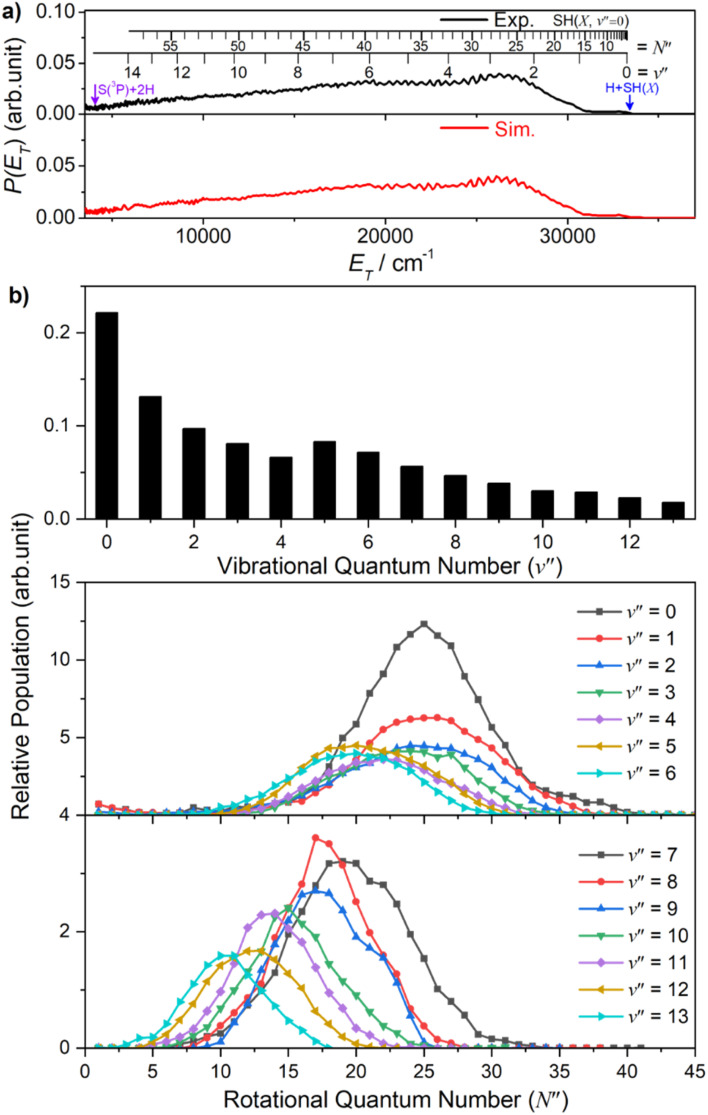
(a) The simulation (red) of the experimental (black) *P*(*E*_T_) spectrum derived from the H atom TOF spectrum following photodissociation of H_2_S at *λ* = 154.53 nm, measured along a detection axis aligned at θ = 54.7° to the ***ε*** vector of the photolysis laser radiation, together with (b) the SH(*X*) vibrational and rotational level population distributions derived therefrom.

The onset at *E*_T_ ∼ 33 000 cm^−1^ (Fig. 2(a)) confirms formation of some internally ‘cold’ SH(*X*) products (*i.e.* SH(*X*) fragments with *v*′′ = 0 and low *N*′′), and the spacing of the partially resolved structure in the range 28 000 ≥ *E*_T_ ≥ 20 000 cm^−1^ can only be accommodated by invoking contributions from SH(*X*) fragments with low *v*′′ and much higher *N*′′ (∼20–30). Such a bimodal rotational state population distribution is reminiscent of that reported previously when exciting at *λ* = 157.6 nm.^48^Fig. 3 shows the simulation of the *P*(*E*_T_) distribution resulting from photolysis at *λ* = 154.53 nm with ***ε*** aligned at the magic angle (θ = 54.7°) to the detection axis, and the SH(*X*, *v*′′, *N*′′) population distributions derived therefrom. The vibrational state population distribution, *P*(*v*′′), maximizes at *v*′′ = 0 and declines monotonically out to at least *v*′′ = 13. The *P*(*N*′′) distributions for all *v*′′ peak at *N*′′ ≫ 0, but the peaks of the various *N*′′ distributions do show a systematic shift to lower *N*′′ with increasing *v*′′.

The *P*(*E*_T_) spectra obtained following photolysis of H_2_S at shorter wavelengths reveal similarly extensive SH(*X*, *v*′′, *N*′′) product state distributions and fragment recoil velocities that preferentially align perpendicular to ***ε***, but with obvious wavelength dependent differences. For example, the relative yield of SH(*X*, low *E*_int_) products formed at *λ* = 151.64 nm or 149.15 nm (Fig. S2(b) and (c)†) is greater than that at *λ* = 154.53 nm, but the clarity of the partially resolved structure in the range 8000 ≤ *E*_int_ ≤ 17 000 cm^−1^ again indicates a significant yield of much more highly rotationally excited SH(*X*), low *v*′′ fragments. Fig. S2(b)† also shows a progression in the range 20 000 ≤ *E*_int_ ≤ 27 000 cm^−1^ attributable to highly vibrationally excited SH(*X*) products with *v*′′ in the range 9–13 carrying less rotational excitation. The simulations of these spectra (Fig. S5 and S6†) return similarly broad *P*(*v*′′) distributions to that found at *λ* = 154.53 nm, stretching to *v*′′ ∼ 13, with associated *P*(*N*′′) distributions that peak well away from *N*′′ = 0.

There is an obvious step change in the low *E*_T_ part of spectra recorded at all shorter excitation wavelengths. The *P*(*E*_T_) spectra obtained at *λ* = 149.15 nm (Fig. 2(b)) again show extensive structure associated with formation of SH(*X*, *v*′′, *N*′′) products in a broad range of quantum states, including features in the range 10 000 ≥ *E*_T_ ≥ 7000 cm^−1^ that can only be attributed to SH(*X*) products with low *v*′′ and exceptionally high *N*′′ quantum numbers. But Fig. 2(b) also shows an obvious new feature: the sharp step at *E*_T_ = *E*_phot_ − *E*_th_(2), where *E*_th_(2) is the threshold energy for forming H + SH(*A*) products. These SH(*A*) fragments are formed mainly in their *v*′ = 0 state, but in a spread of rotational levels extending to the limit dictated by overall energy conservation (*i.e.* to *E*_T_ ∼ 0). By extrapolation from the data taken at longer wavelengths, this structure associated with H + SH(*A*) fragments is likely to be riding on top of more continuous signal associated with the three-body dissociation process (3). It will be further contaminated by secondary H atoms formed (in conjunction with S(^3^P_*J*_) atoms) by predissociation of the primary SH(*A*) fragments.^51^ Every primary SH(*A*) photofragment arising in the photodissociation of H_2_S will dissociate to yield a second H atom within a few nanoseconds and these secondary H atoms inevitably contribute to the measured H atom TOF spectra. The predissociation of SH(*A*) radicals favours population of the ground (*J* = 2) spin–orbit state of the S(^3^P_*J*_) products,^51^ and combs indicating the expected *E*_T_ values of H + S(^3^P_2_) products from predissociation of SH(*A*, *v*′ = 0, *N*′) levels populated in the photolysis of H_2_S at *λ* = 139.11 nm are included in the inset in Fig. 2(c).

The *P*(*E*_int_) spectra obtained at *λ* = 146.30 nm (Fig. S2†(d)) and at 143.15, 140.89, 139.11 and 133.26 nm (Fig. S3†) reveal broadly similar behaviour, *i.e.* formation of SH(*X*) products in a broad, wavelength dependent range of *v*′′, *N*′′ levels and increasingly dominant H + SH(*A*) product yields. Note that the highest SH(*A*, *v*′ = 0, *N*′) levels observed at *λ* = 139.11 nm (Fig. 2(c)) and at all shorter wavelengths have rotational energies greater than the bond dissociation energy of the SH(*A*) state – shown by the arrow indicating the energetic threshold for 2H + S(^1^D) product formation (process (4)). The signal at lowest *E*_T_ in Fig. 2(c) will also include any contributions from direct three-body-dissociation to such products. Population of such SH(*A*) ‘super-rotor’ levels has been reported previously following H_2_S photolysis at much shorter wavelengths^49,50^ and for both OH(*X*) and OH(*A*) fragments from suitably short wavelength photolysis of H_2_O.^52,53^

**Fig. 4:**
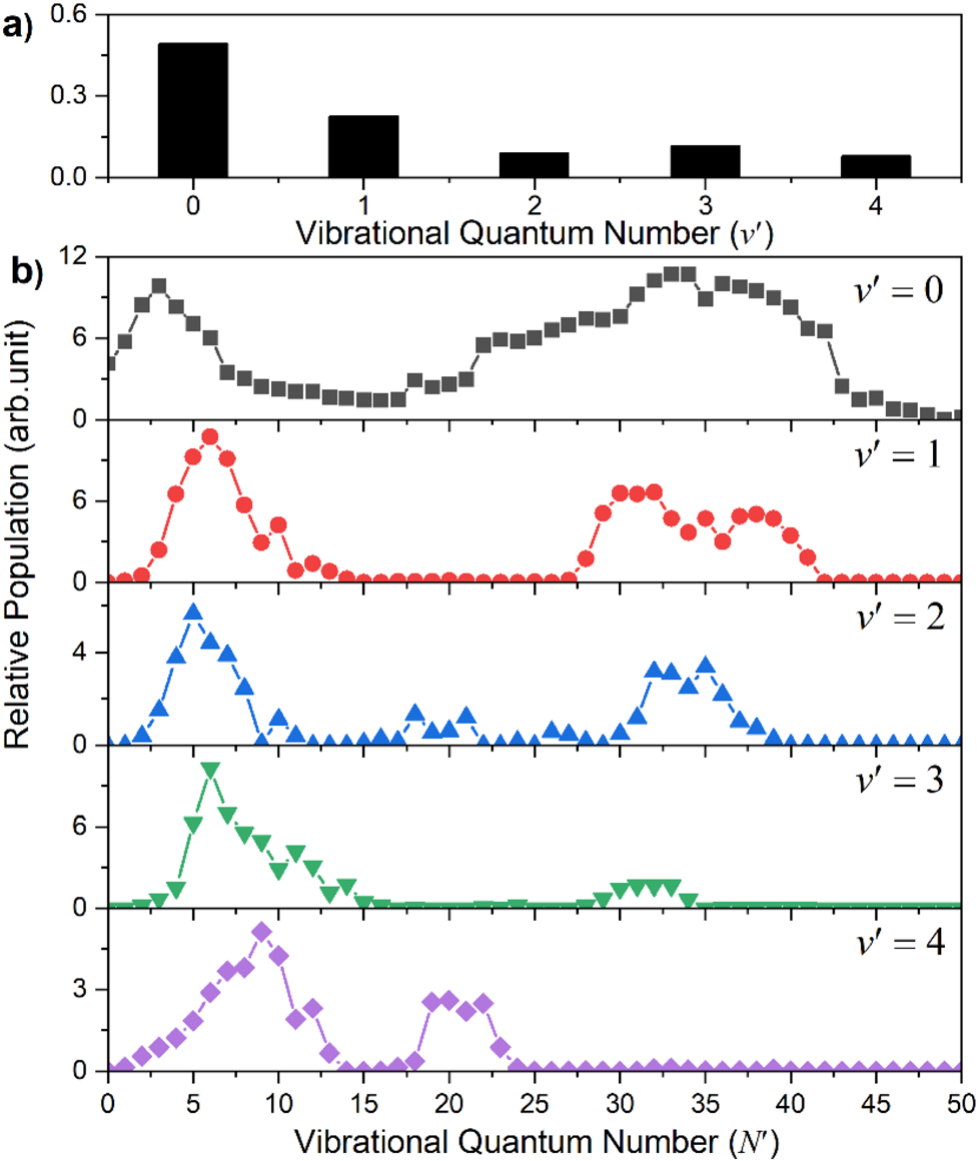
The SH(*A*) vibrational (a) and rotational (b) state population distributions following photodissociation of H_2_S at *λ* = 122.95 nm.

Similar trends persist at all shorter photolysis wavelengths, as illustrated by the *P*(*E*_T_) data recorded at *λ* = 122.95 nm (Fig. 2(d)) and by the *P*(*E*_int_) spectra obtained at *λ* = 131.32, 129.95, 129.12, 125.05 and 122.95 nm shown in Fig. S4.† These show H + SH(*A*) photoproducts becoming increasingly dominant at shorter wavelengths and that the SH(*A*) products are formed in a broad range of *E*_int_ states, including ‘super rotor’ levels. However, the detailed forms of the spectra show marked variations with photolysis wavelength – with respect to both the SH(*A*) quantum state population distributions and the relative yields of SH(*A*) and SH(*X*) fragments – as can be seen by contrasting *P*(*E*_int_) spectra obtained at two nearby wavelengths: *λ* = 129.95 and *λ* = 129.12 nm (Fig. S4(b) and (c)†). The ‘steps’ indicating formation of SH(*A*) fragments with *v*′ = 0 and 1 and low *N*′ are much more evident in the former, whereas the extended feature attributable to H + SH(*X*) products is only really evident in the latter. Fig. 4 shows the best estimate *P*(*v*′) and *P*(*N*′) distributions in the primary SH(*A*) products following excitation at *λ* = 122.95 nm, the fitting procedure is shown in Fig. S7.† The overall trends – namely that the *P*(*v*′) distribution declines with increasing *v*′ and that each *P*(*N*′) distribution is bimodal, involving both rotationally ‘cold’ and rotationally ‘hot’ fractions – is considered robust. These various observations, especially the bimodal quantum state distributions of SH(*X*) and SH(*A*) fragments will be discussed later.

### (b) Imaging S(^1^D) photoproducts

**Fig. 5:**
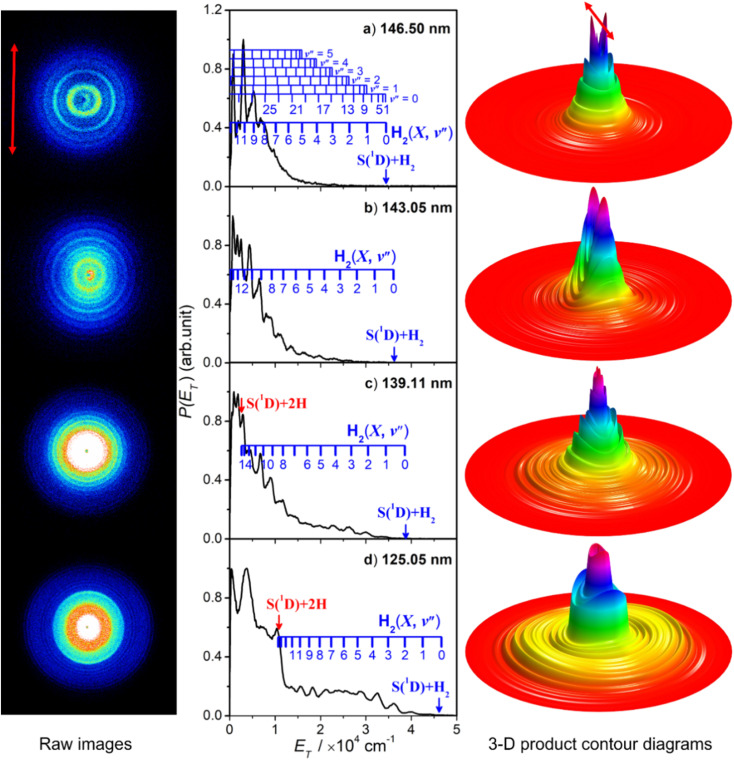
The time-sliced velocity map images of the S(^1^D) photofragments from photolysis of H_2_S at *λ* = (a) 146.50 nm, (b) 143.05 nm, (c) 139.11 nm and (d) 125.05 nm, with the ***ε*** vector of the photolysis laser radiation (shown by the double headed red arrow on the top left image) aligned vertically in the plane of the image. The corresponding 3-D product contour, *P*(θ,*E*_T_), diagrams are shown to the far right of each row, with the ***ε*** vector again indicated by the red arrow on the top diagram. The centre columns show the *P*(*E*_T_) distributions derived from analysis of these images (assuming H_2_ as the partner fragment), with superposed combs showing the H_2_(*v*′′) and (in panel (a) the H_2_(*v*′′, *J*′′)) states responsible for the evident structure. The navy and red vertical arrows indicate the maximum *E*_T_ values associated with, respectively, channels (5) and (4).

Fig. 5 shows time-sliced velocity map images of the S(^1^D) photofragments formed by photolysis of H_2_S at *λ* = (a) 146.50 nm, (b) 143.05 nm, (c) 139.11 nm and (d) 125.05 nm. The corresponding *P*(*E*_T_) spectra were derived assuming that the recoiling co-fragment is an H_2_ molecule. The false colour *P*(θ,*E*_T_) plots shown at the right of the figure illustrate the wavelength- and *E*_T_-dependent recoil anisotropy. The results obtained at other wavelengths in the range 140.96 ≥ *λ* ≥ 121.60 nm are shown in Fig. S8.†

Attempts to record S(^1^D) atom images when exciting parent resonances at *λ* > 146.50 nm were unsuccessful. The energy of a *λ* = 146.50 nm photon is insufficient to access the three-body fragmentation process (4), so the co-fragments in the image recorded at this wavelength (Fig. 5(a)) and at *λ* = 143.05 nm (Fig. 5(b)) must all be ground (*X* state) H_2_ molecules. The combs superposed over the *P*(*E*_T_) spectra show the *E*_T_ values predicted for the various H_2_ quantum states.

**Fig. 6:**
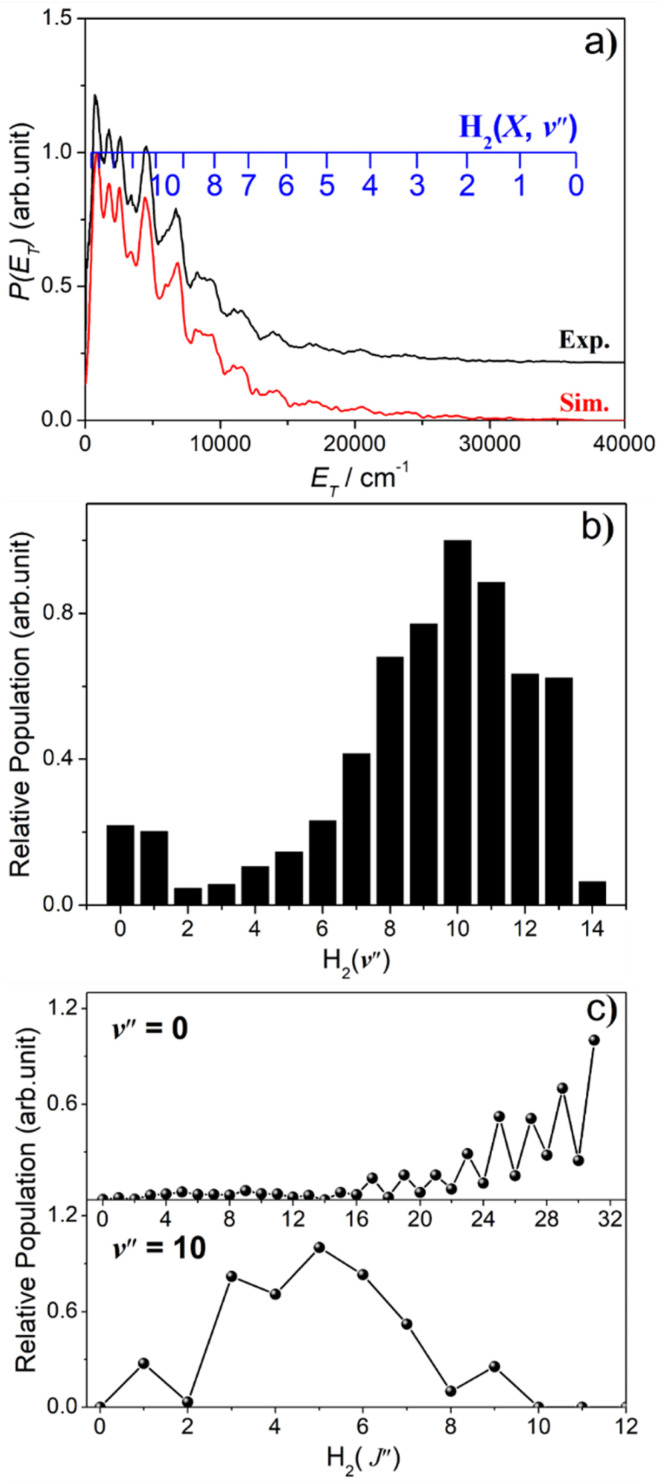
(a) The best-fit simulation (red) of the experimental *P*(*E*_T_) spectrum (black) derived from the S(^1^D) fragment ion image measured following photodissociation of H_2_S at *λ* = 143.05 nm. The latter has been offset vertically for display purposes. (b) The H_2_ vibrational and (c) selected rotational level population distributions derived therefrom.

The simulation of the *P*(*E*_T_) spectrum at *λ* = 143.05 nm (Fig. 6) reveals a bimodal distribution of H_2_(*v*′′, *J*′′) products. Most H_2_ fragments are formed highly vibrationally excited (with a most probable vibrational quantum number *v*′′_mp_ ∼ 10) but with only modest rotational excitation (*J*′′_mp_ ∼ 5). A small fraction shows a completely different energy disposal, however, involving minimal vibration excitation (*v*′′_mp_ = 0, 1) and a highly inverted rotational population distribution that appears to peak at the highest *J*′′ value permitted by energy conservation. The finding of highly rovibrationally excited H_2_ fragments accords with the conclusions of a previous one colour resonance enhanced multiphoton ionization (REMPI) study of the H_2_ fragments formed when exciting H_2_S (using two-photons) to similar total energies,^54^ and we note that Ubachs and co-workers^55–57^ have recently exploited similar two-photon excitations of H_2_S as a source for high resolution spectroscopy studies of highly excited rovibrational levels of H_2_.

The simulations of the *P*(*E*_T_) distributions obtained at *λ* = 146.50 and 139.11 nm (Fig. S9†) return similar bimodal H_2_ product state population distributions but, even by *λ* = 140.96 nm (Fig. S8(a)†), the rival three-body channel (4) is starting to contribute to the measured S(^1^D) images. This introduces further uncertainty in simulating the *P*(*E*_T_) spectra, and the best-fit simulation shown for *λ* = 125.05 nm (Fig. S9†) is guided by assuming two contributing energy disposals in the S(^1^D) + H_2_(*v*′′, *J*′′) products as at longer excitation wavelengths. For completeness, Fig. S10† shows H_2_(*v*′′), H_2_(*v*′′ = 0, *J*′′) and H_2_(*v*′′ = 10, *J*′′) product state population distributions at the five other photolysis wavelengths.

Consideration of the H and S(^1^D) atom PTS data in tandem indicates that the quantum yield for forming S(^1^D) atoms in the VUV photolysis of H_2_S is small. Each S(^1^D) atom contributing to the dominant central features in the images shown in, for example, Fig. 5(d) is accompanied by two H atoms which should be evident at low *E*_T_ in the corresponding *P*(*E*_T_) spectra derived from the H atom TOF measurements. But the *P*(*E*_T_) distributions derived from the H atom TOF measurements at these same short excitation wavelengths show no noticeable discontinuity at low *E*_T_ values, reinforcing the view that H–SH bond fission is the dominant primary fragmentation pathway in the VUV photodissociation of H_2_S.^26^

The recoil velocity distributions of the S(^1^D) products revealed in Fig. 5 and S8† are relatively isotropic, but closer inspection of the various *P*(θ,*E*_T_) plots reveal subtle wavelength dependent variations in recoil anisotropy. All S(^1^D) + H_2_(*v*′′, *J*′′) products formed at *λ* = 146.5 nm show preferentially perpendicular recoil anisotropy as, more weakly, do those formed at *λ* = 139.11 nm. Those formed at *λ* = 143.05 nm, however, show a preference for recoil parallel to ***ε***. As Fig. 5(d) shows, the *λ* = 125.05 nm data display both behaviours: the faster S(^1^D) atoms show preferential perpendicular recoil anisotropy, while the slower S(^1^D) atoms favour parallel recoil. In all cases where the photon energy is sufficient to allow atomization (4), the recoil anisotropies of the slower S(^1^D) atoms appear the same, whether formed along with two H atoms or with H_2_(high *E*_int_) partners, suggesting that the atomization revealed *via* the S(^1^D) imaging studies should be regarded as extrapolations of the S(^1^D) + H_2_ formation dynamics.

### (c) Imaging S(^1^S) photoproducts

**Fig. 7:**
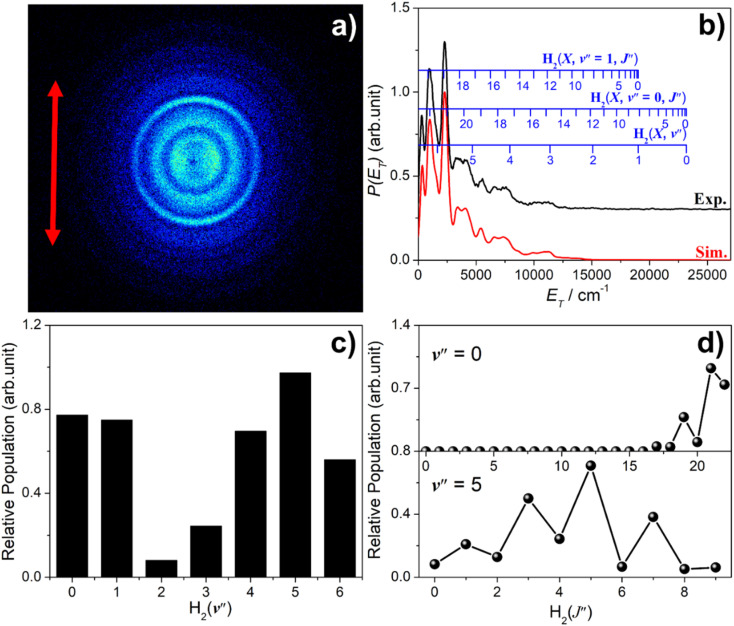
(a) Time-sliced velocity map image of the S(^1^S) photofragments from photolysis of H_2_S at *λ* = 143.00 nm, with the ***ε*** vector of the photolysis laser radiation (shown by the double headed red arrow) aligned vertically in the plane of the image. (b) The *P*(*E*_T_) distribution derived from this image (black), assuming H_2_ as the partner fragment, along with best-fit simulation (red), offset vertically for clarity. The superposed combs show the *E*_T_ values associated with H_2_ (*v*′′, *J*′′ = 0), (*v*′′ = 0, *J*′′) and (*v*′′ = 1, *J*′′) states that contribute to the evident structure. (c) The H_2_ vibrational and (d) selected H_2_(*v*′′) rotational level population distributions from the best-fit simulation.

Images of S(^1^S) atom products arising from photolysis of H_2_S were also recorded, with resonant ionization detection using *λ* = 136.13 nm photons. A wavelength-dependent study of S(^1^S) production in the photolysis of H_2_S at shorter wavelengths has recently appeared elsewhere.^58^ As in the case of S(^1^D) products, attempts to detect S(^1^S) atoms following photolysis at *λ* ≥ 146.50 nm were unsuccessful. The threshold energy for process (6) lies well below that provided by a *λ* = 143.00 nm photon, so the structure in the image and the accompanying *P*(*E*_T_) distribution necessarily reflects population of different *v*′′, *J*′′ states of the H_2_ partner fragment. Fig. 7 shows the simulation of the *P*(*E*_T_) distribution obtained at *λ* = 143.00 nm, which returns a bimodal H_2_ product state population distribution involving both high *v*′′, low *J*′′ and low *v*′′, high *J*′′ components, reminiscent of that observed for the corresponding S(^1^D) + H_2_ products, but the high *v*′′ (*v*′′ ∼ 3–6), low *J*′′ becomes dominant as the excitation wavelength is reduced.^58^

Estimating the quantum yields of fragmentation channels probed in different PTS experiments is complicated by hard to define detection efficiencies, but the current knowledge suggests that the S(^1^S) + H_2_ yield is lower than that for S(^1^D) + H_2_ products.^58^ Both constitute a photochemical source of vibrationally excited H_2_ molecules when H_2_S molecules are exposed to the general interstellar radiation field (ISRF).

### (d) Electronic structure calculations

The foregoing PTS data offer an unprecedently wide-ranging and detailed view of the primary photochemistry of H_2_S at excitation energies up to the onset of ionization. Interpretation of such data requires a detailed knowledge of the excited state PESs that guide the fragmentation dynamics. This section reports results of extensive new *ab initio* data for the first few singlet states of H_2_S that allow deeper interpretation of the experimental data.

The vertical excitation energies (VEEs) from the ground (1^1^A′) state equilibrium geometry to the first few singlet excited states of ^1^A′ and ^1^A′′ symmetries are shown in Table 1. The VEEs calculated in the present work using both multi-reference configuration interaction (MRCI) methods (Table 1) and by equation-of-motion coupled cluster single and double excitation (EOM-CCSD) methods (Table S1†) are reassuringly consistent with previous studies.^59–61^ The present calculations are equally successful at replicating the various documented H + SH, H_2_ + S and 2H + S dissociation energies, as shown in Table S2.†

**Table 1:** The vertical excitation energies (VEEs) to the first few excited states of H_2_S with ^1^A′ and ^1^A′′ symmetries, from the ground state equilibrium geometry, along with the dominant orbital promotion. The later columns list VEEs from selected previous *ab initio* theory and one-photon spectroscopy studies, and the assignments of experimentally observed features proposed in the present work

Electronic state	Orbital promotion	VEE/eV	Previous theory/eV (ref.)			Experiment/eV (ref.)	
			RPA^59^	MRD-CI^60^	MR-SDCI^61^	1-Photon	Proposed
1^1^A_1_ (1^1^A′)	—	0	0	0	0		
2^1^A_1_ (2^1^A′)	3b_1_ ← 2b_1_	8.22	8.32	7.97	8.24	8.03 (ref. 43)	8.19 (ref. 66)
1^1^B_2_ (3^1^A′)	1a_2_ ← 2b_1_	8.74	9.03	8.69	8.70	8.80,^43^ 8.79 (ref. 72)	
3^1^A_1_ (4^1^A′)	4b_1_ ← 2b_1_	8.84	9.05	8.70	8.81	8.80 (ref. 73)	8.81 (ref. 66)
4^1^A_1_ (5^1^A′)	6a_1_ ← 5a_1_	9.27	9.39	9.04	9.33	9.27 (ref. 43)	9.34 (ref. 66)
1^1^A_2_ (1^1^A′′)	3b_2_ ← 2b_1_	6.15	6.54	6.80	6.33	4.6–7.5 (ref. 43)	
1^1^B_1_ (2^1^A′′)	6a_1_ ← 2b_1_	6.23	6.57	6.16	6.47	6.33,^43^ 4.59–6.52 (ref. 73)	
2^1^A_2_ (3^1^A′′)	4b_2_ ← 2b_1_	7.89	8.08	7.70	7.98	7.85 (ref. 43)	7.89 (ref. 65 and 66)
2^1^B_1_ (4^1^A′′)	7a_1_ ← 2b_1_	8.00	8.16	7.80	8.10	8.18–8.32 (ref. 43)	8.03 (ref. 66)
3^1^B_1_ (5^1^A′′)	8a_1_ ← 2b_1_	8.47	8.75	8.40	8.49	8.84,^43^ 8.66 (ref. 45)	

#### H–SH bond fission

**Fig. 8:**
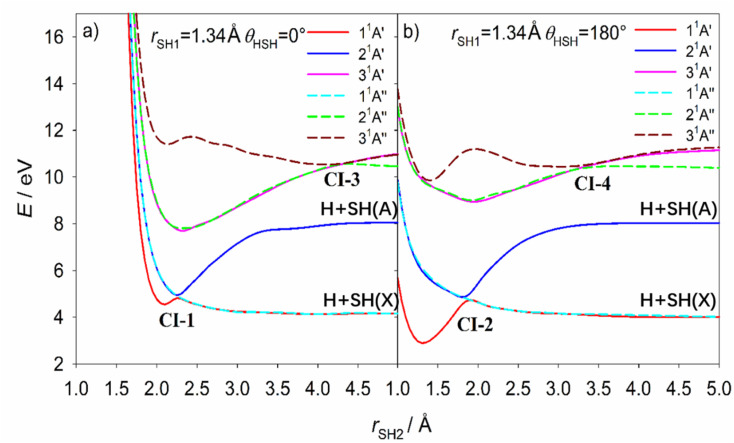
The cuts through the calculated PESs for the three lowest energy states of H_2_S with ^1^A′ symmetry and the three lowest energy states with ^1^A′′ symmetry at linear (a) H⋯HS and (b) H⋯SH geometries (*θ* = 0 and 180°, respectively), plotted as a function of one S–H bond length, *r*_SH2_, with the other held fixed at its ground state equilibrium value (*r*_SH1_ = 1.34 Å). The conical intersections (CIs 1–4) have been marked in the figures. The adiabatic 1^1^A′ and 1^1^A′′ PESs correlate with ground state H + SH(*X*) products while the adiabatic 2^1^A′ PES correlates to the excited H + SH(*A*) dissociation limit at bent geometry. All energies are defined relative to the ground state minimum energy geometry at *E* = 0.

Accurate prediction of the VEEs and the corresponding transition dipole moments (TDMs) are necessary but not sufficient for understanding the fragmentation dynamics, which reflect the topographies of the multi-dimensional PES(s) sampled following vertical excitation, *en route* to eventual dissociation. The following discussion focuses mainly on the three lowest-lying ^1^A′ PESs and the three lowest-lying ^1^A′′ PESs. Fig. 8 shows cuts through the PESs for these six states of H_2_S at linear H⋯HS and H⋯SH geometries (*θ* = 0 and 180° respectively), plotted as a function of one S–H bond length, *r*_SH2_, with the other fixed at its ground state equilibrium value (*r*_SH1_ = 1.34 Å). These plots illustrate the degeneracy of the 2^1^A′ and 1^1^A′′ excited states at short *r*_SH2_ and linear geometries, and the conical intersections between the 2^1^A′ and 1^1^A′ PESs at linear H⋯HS and H⋯SH geometries (labelled as CI-1 and CI-2 in Fig. 8). The geometries for the minimum energies of these and other CIs (the MECIs) identified in the present work are listed in Table 2. Analogous cuts through these potentials at other non-linear geometries (*θ* = 30, 60, 90, 120 and 150°, Fig. S11†) show that the energies of the 2^1^A′ and 1^1^A′′ states (which are the two Renner–Teller components of a ^1^Π state) split apart on bending, and that at bent geometries the adiabatic 1^1^A′ and 1^1^A′′ PESs correlate with ground state H + SH(*X*) products while the adiabatic 2^1^A′ PES correlates to the excited H + SH(*A*) dissociation limit. Fig. 8 also illustrates that the 3^1^A′ and 2^1^A′′ states are the degenerate Renner–Teller components of a ^1^Δ state at linear geometries and short *r*_SH2_, and reveals conical intersections (CI-3 and CI-4) between the 2^1^A′′ and 3^1^A′′ potentials at extended *r*_SH2_ at *θ* = 0° and at *θ* = 180°. In addition, the 1^1^A′′ and 2^1^A′′ PESs and the 2^1^A′ and 3^1^A′ PESs show CIs (here labelled CI-5 and CI-6, respectively) in the near-vertical region, which can be recognised by the potential energy cuts for *θ* = 90° in Fig. S11.† The 3-D representations of the PESs highlight these more clearly and reveal other important regions of CIs between these various PESs, as shown in Fig. S12.†

**Table 2:** The calculated geometries at the minimum energies (*E*, defined relative to the global minimum of the ground state) of the conical intersections (MECIs) identified by the present *ab initio* calculations

CI		*θ*/°	*r*_SH1_/Å	*r*_SH2_/Å	*E*/eV
1^1^A′–2^1^A′	CI-1	0	1.74	2.60	4.45
	CI-2	180	1.36	1.85	4.68
2^1^A′′–3^1^A′′	CI-3	0	1.55	4.16	9.70
	CI-4	180	1.64	2.60	9.35
1^1^A′′–2^1^A′′	CI-5	95	1.33	1.33	6.23
2^1^A′–3^1^A′	CI-6	91	1.58	1.58	8.82

CI-5, between the 1^1^A′′ and 2^1^A′′ PESs, has been reported previously and the MECI geometry (Table 2) is sensibly consistent with that proposed by Simah *et al.*^62^ The seam of intersection between these two PESs spans a wide range of bond angles and bond lengths, however, as shown in Fig. S13 and S14.† Recognizing the potential interference between the 1^1^A′′ ← 1^1^A′ and 2^1^A′′ ← 1^1^A′ transition amplitudes and the predissociating quasi-bound resonances supported by the 2^1^A′′ PES were key to describing the diffuse structure evident in the H_2_S long wavelength absorption continuum^62,63^ and explaining the increasing vibrational excitation of the SH(*X*) products observed when exciting at shorter wavelengths within this continuum.^46,48^ CI-6 has not been detailed previously but, given that it lies at near vertical geometries, it surely offers a non-adiabatic pathway for funnelling population between the 3^1^A′ and 2^1^A′ PESs – a point to which we return in the next section.

The contour plots for the 1^1^A′, 2^1^A′, 3^1^A′, 1^1^A′′, 2^1^A′′, 3^1^A′′ and 4^1^A′′ potentials shown in Fig. S15† allow further scrutiny of the complex and varying topographies of the respective PESs. The 2^1^A′ PES displays a particularly rich topography, including a shallow minimum in the vertical region and seams of intersection and conical intersections with the lower lying ^1^A′′ and 1^1^A′ states at *θ* = 0 and 180°. The 3^1^A′, 2^1^A′′, 3^1^A′′ and 4^1^A′′ PESs each display more pronounced local minima in the vertical region, indicating significant Rydberg character in each case. The predicted depths of these minima would each be sufficient to support several (quasi-)bound levels.

#### S atom elimination

**Fig. 9:**
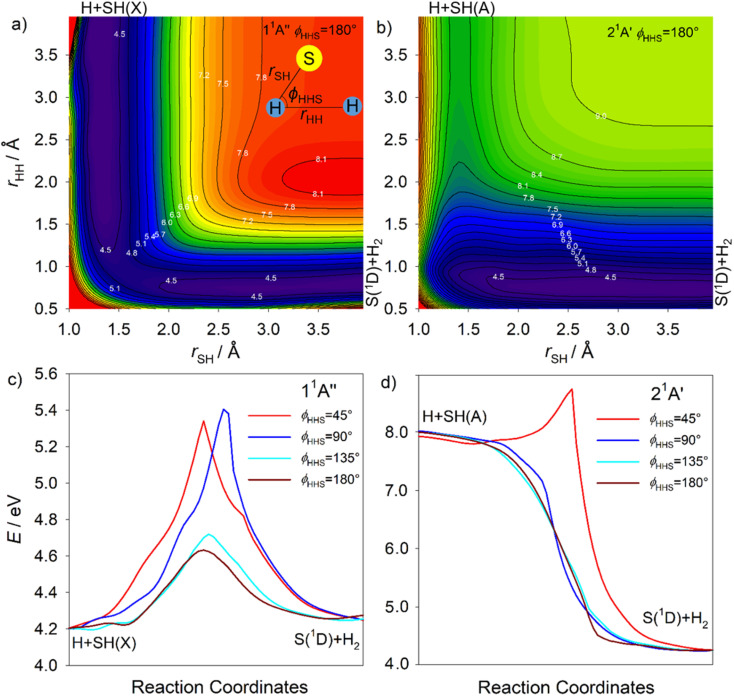
The false colour plots showing the (a) 1^1^A′′ and (b) 2^1^A′ PESs as functions of the H–H distance (*r*_HH_) and the shorter S–H separation (*r*_SH_) for an ∠HHS bond angle *ϕ*_HHS_ = 180°. The panels (c) and (d) show the respective minimum energy paths (MEPs) along the H + SH → S + H_2_ reaction coordinate, for *ϕ*_HHS_ = 45, 90, 135 and 180°. The energy contours are labelled in eV, defined relative to the ground state minimum energy geometry at *E* = 0.

Theory is also able to inform on fragmentation pathways that likely contribute to the experimentally observed S(^1^D) and S(^1^S) product yields. Fig. 9(a) and (b) show contour plots for, respectively, the 1^1^A′′ and 2^1^A′ PESs as functions of the H–H distance (*r*_HH_) and the shorter S–H separation (*r*_SH_), for an ∠HHS bond angle *ϕ*_HHS_ = 180°. These data are reproduced along with equivalent plots for *ϕ*_HHS_ = 0, 45, 90 and 135° in Fig. S16 and S17.† As Fig. 9(c) and (d) show, the calculations return very different minimum energy paths (MEPs) along the H + SH → S + H_2_ reaction coordinate on these two PESs. The MEP from H + SH(*A*) to S(^1^D) + H_2_ products on the 2^1^A′ PES is exoergic and barrierless at all but small *ϕ*_HHS_.^64^ The H + SH(*X*) → S(^1^D) + H_2_ MEP on the 1^1^A′′ PES, in contrast, is mildly endothermic, and shows a minimum barrier height of ∼0.4 eV at *ϕ*_HHS_ = 180° which increases to >1 eV by *ϕ*_HHS_ = 90°.

Fig. S18† shows alternative depictions of the 1^1^A′′, 2^1^A′′, 2^1^A′ and 3^1^A′ PESs, plotted using Jacobi coordinates, with *R* defining the distance between the S atom and the H_2_ centre of mass and *r*_HH_ denoting the H–H separation, for an S–H_2_ Jacobi angle *ϕ*_J_ = 90°. These plots illustrate the change in *r*_HH_ induced by the approaching S(^1^D) atom. All show the expected minimum at large *R* and *r*_HH_ ∼ 0.8 Å (bottom right-hand corner) associated with S(^1^D) + H_2_ products. The 1^1^A′′ and 2^1^A′ PESs both hint at the minimum at linear geometries (*R* → 0 and *r*_HH_ ∼ 2.7 Å, top left-hand corner). The minimum in the vertical region of the 2^1^A′′ PES is obvious (at *R* ∼ 0.93 Å, *r*_HH_ ∼ 1.93 Å, marked by the white dot in each panel), as are shallower minima in this region of both the 2^1^A′ and 3^1^A′ PESs. The barrier to S(^1^D) elimination following vertical excitation to the 1^1^A′′ PES is negligible, and clearly much smaller than that for dissociation on the 2^1^A′′ PES. S(^1^D) elimination following vertical excitation to the 2^1^A′ and 3^1^A′ PESs is also predicted to be essentially barrierless.

**Fig. 10:**
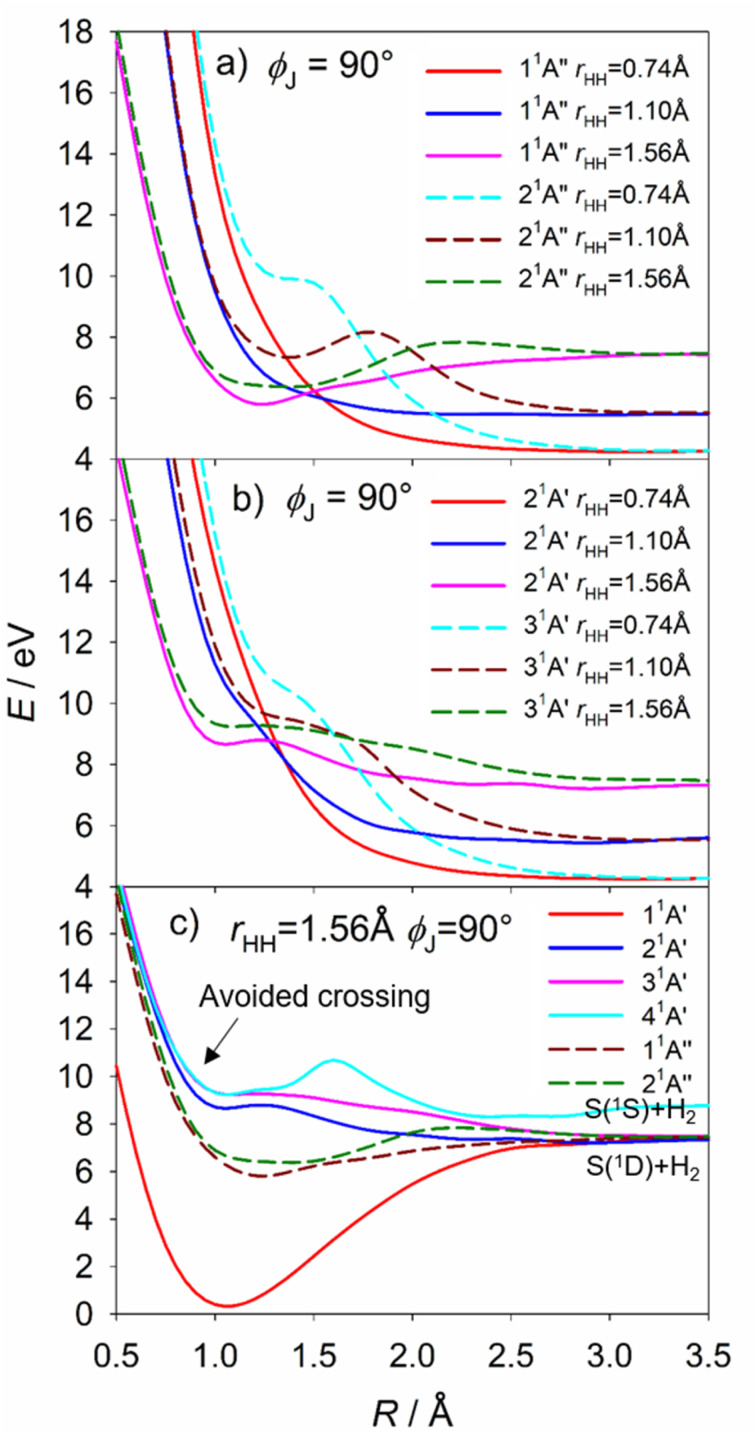
The cuts through the S–H_2_ potentials for the (a) 1^1^A′′ and 2^1^A′′ and (b) 2^1^A′ and 3^1^A′ states, as functions of *R*, for *r*_HH_ = 0.74, 1.10 and 1.56 Å (*i.e.* reasonable average bond lengths for H_2_ molecules with, respectively, *v* = 0, ∼5 and ∼10) and *ϕ*_J_ = 90°. (c) The *ϕ*_J_ = 90° cuts for the five states that correlate to the S(^1^D) + H_2_ dissociation limit and the 4^1^A′ potential linking to the S(^1^S) + H_2_ limit. There is an avoided crossing between the 4^1^A′ and 3^1^A′ potentials. All energies are defined relative to the ground state minimum energy geometry at *E* = 0.

The S(^1^D) imaging experiments showed an obvious propensity for forming vibrationally excited H_2_ co-fragments. To inform on this process, Fig. 10(a) and (b) show cuts through the S–H_2_ potentials for the ^1^A′′ and ^1^A′ excited states featured in Fig. S18,† as a function of *R*, for three different fixed *r*_HH_ values (*r*_HH_ = 0.74, 1.10 and 1.56 Å, which are representative average bond lengths for H_2_ molecules with, respectively, *v*′′ = 0, ∼5 and ∼10) and a Jacobi angle *ϕ*_J_ = 90°. These serve to reinforce the conclusion that the 1^1^A′′, 2^1^A′ and 3^1^A′ PESs all present negligible barrier to S(^1^D) elimination. The *ϕ*_J_ = 90° cuts for these four states are reproduced again in Fig. 10(c), along with those for the 1^1^A′ and 4^1^A′ states, to show the correlations between the parent states and the S(^1^D) + H_2_ and S(^1^S) + H_2_ dissociation limits. Optimized geometries and energies of the transition states along both the H + SH and S + H_2_ dissociation pathways for each of these electronic states are listed in Table S3.† Fig. S19† shows another cut through the 3^1^A′ and 4^1^A′ potentials, which reveals an avoided crossing between these PESs that likely facilitates non-adiabatic coupling to the observed S(^1^S) + H_2_ product channel.

### (e) The photofragmentation dynamics

#### Parent spectroscopy

The primary aim of this work was to gain a comprehensive picture of the wavelength-dependent photofragmentation dynamics of H_2_S, but the study also affords new insights into the VUV absorption of H_2_S and, particularly, allows us to comment on the recognized surfeit of absorption features in the 148–158 nm range. Quantum defect arguments encourage the expectation that this wavelength region should be dominated by transitions to the 4p-Rydberg complex.^43,45^ The present data (Table 1) confirm previous theoretical conclusions^59–61^ that the energetic ordering of the three 4p ← 2b_1_ Rydberg origins is ^1^A_2_ < ^1^B_1_ < ^1^A_1_. The traditional assignments from the one photon absorption study, however, suggest the reverse ordering of the ^1^B_1_ and ^1^A_1_ states.^43^ We now seek a resolve this discrepancy.

The 2 + 1 and 3 + 1 REMPI studies of H_2_S and D_2_S confirmed the origin of the 2^1^A_2_ (4b_2_ ← 2b_1_) state at 7.89 eV,^65,66^ near coincident with a weak feature in the one photon spectrum^44^ and in excellent accord with the current theoretical prediction (Table 1). As Table S1† shows, the 2^1^A_2_–*X̃*^1^A_1_ transition is one-photon forbidden in the *C*_2v_ group, but it could gain some transition strength by coupling with near resonant states of ^1^B_1_ symmetry. The 2 + 1 REMPI spectroscopy revealed another feature with an origin at 8.19 eV, with a very simple band contour, that disappeared when the polarization of the exciting radiation was switched from linear to circular.^66^ Such behaviour is only consistent with a totally symmetric excitation and the feature was logically assigned to the 2^1^A_1_–*X̃*^1^A_1_ (3b_1_ ← 2b_1_) transition. The non-recognition of this transition in one photon absorption can be explained by its small oscillator strength, which reflects the fact that the donor (2b_1_) and acceptor (3b_1_) orbitals both have predominant ‘atomic p-like’ character in the vertical region.

The present calculations place the 2^1^B_1_ state between the 2^1^A_2_ and 2^1^A_1_ states and predict a 2^1^B_1_–*X̃*^1^A_1_ oscillator strength more than one order of magnitude greater than that for the 2^1^A_1_–*X̃*^1^A_1_ transition. The prominent absorption band at *λ* ∼ 154.5 nm, hitherto assigned as a ^1^A_1_–*X̃*^1^A_1_ transition,^43^ is thus most plausibly associated with the ^1^B_1_ member of the 4p-complex (*i.e.* the 2^1^B_1_ state). This absorption feature appears only to have been measured under sufficiently saturated conditions to mask any useful insights from its band contour but its energy, *E* ∼ 8.03 eV, again matches very well with theory (Table 1). Most of the recognized weaker absorption bands in the 148–154 nm region have been shown to display band contours consistent with a ^1^B_1_ state assignment. Thus, based on the currently available data, it is logical to assign these features to transitions to predissociating excited vibronic levels of two ^1^A′′ states.

#### Photofragmentation dynamics

For completeness, we start by noting that the previous PTS studies of H_2_S exciting at *λ* ≥ 198 nm observed H + SH(*X*, low *v*′′, low *N*′′) products, with perpendicular recoil anisotropies.^46^ Such energy disposal is consistent with direct excitation to the coupled 1^1^A′′/2^1^A′′ continuum (*i.e.* excitation *via* a TDM aligned perpendicular to the molecular plane) and dissociation on a timescale that is short compared to that of parent rotation.

The *λ* ∼ 150–160 nm region shows the first Rydberg resonances. Dissociation following excitation of the resonances at *λ* = 154.53 and 151.64 nm yields H atoms together with SH(*X*) fragments in very wide spreads of *E*_int_ levels, along with some 2H + S(^3^P) products (Fig. 2). The previous PTS study conducted at *λ* = 157.6 nm also identified internally excited SH(*X*) products, though only extending to *v*′′ ∼ 7.^48^ The H + SH(*X*) products in the present studies display perpendicular recoil anisotropy, and no SH(*A*) or S(^1^D) products were identified at either excitation wavelength.

The underlying continuous absorption shows a local minimum in this energy region (Fig. S1†), implying little vertical excitation probability from the ground state to either the 2^1^A′′/1^1^A′′ states responsible for the longer wavelength continuum or to the 2^1^A′ (and 3^1^A′) states that support the background absorption at shorter wavelengths. The excited states that give rise to the resonances in the *λ* ∼ 150–160 nm region have similar minimum energy geometries to the ground state yet manage to achieve sufficient overlap to predissociate by non-adiabatic coupling to one or other or both of these continua. We can envisage two possible explanations for the observed H + SH(*X*) product state distributions. The first attributes the observed energy disposal to the result of coupling to the 2^1^A′′/1^1^A′′ continuum, *i.e.* a fairly simple extrapolation of the behaviour identified at *λ* ∼ 200 nm. Support for this interpretation comes from the prior finding that the 2^1^A_2_ (3^1^A′′) state predissociates homogeneously (*i.e.* by coupling to a continuum of ^1^A′′ symmetry), but we note that one photon excitation to this state is formally forbidden and there must remain some uncertainty as to whether this same state is accessed by photoexcitation at *λ* = 157.6 nm. Nothing in the *ab initio* data indicates how vertical excitation followed by non-adiabatic coupling to the 2^1^A′′ or 1^1^A′′ states would promote such high levels of internal excitation in the SH(*X*) products, though we note that the seam of intersection associated with CI-5 spans a wide range of angles and bond lengths (Fig. S13 and S14†).

Extensive internal excitation of the products of a molecular photodissociation process is more typically associated with evolution on a PES that encourages nuclear motion in coordinates additional to that required for the bond fission. The 2^1^A′ PES of H_2_S shown in Fig. 8 and S15† (the analogues of the *B̃* state potential of H_2_O) has such a topography, which encourages an alternative explanation for the observed formation of SH(*X*) fragments in a wide range of *v*′′ and, particularly, *N*′′ levels. In this scenario, the photo-prepared Rydberg states couple to the low energy region of the 2^1^A′ PES and are accelerated towards linear geometries (*θ* = 180°). The non-adiabatic coupling at the seam of degeneracy with the 1^1^A′′ PES (at *r*_SH2_ bond lengths up to the value at CI-2) or at the conical intersection with the 1^1^A′ PES (CI-2) provides a route to H + SH(*X*) products. This explanation can certainly account for the observed SH(*X*) product rotation.

The available *ab initio* data is less revealing as to the source of the extreme product vibrational excitation, but a potential contributory mechanism is noted at the end of the next paragraph. First, we consider the onset of SH(*A*) product formation. As Fig. 8 showed, the 2^1^A′ PES correlates adiabatically with H + SH(*A*) products. The SH(*A*) products are observed upon tuning to slightly shorter wavelengths (*e.g. λ* = 149.15 nm, Fig. S2(c)†). The SH(*X*) fragments formed at this wavelength display a very similar *P*(*E*_int_) distribution to that found at the longer wavelengths studied. This encourages the view that the topography of the 2^1^A′ PES ensures that molecules coupling to it at energies just above its accession threshold (*e.g.* when exciting at *λ* = 154.53 nm) are unable to dissociate adiabatically (*i.e.* to H + SH(*A*) products) and are instead drawn into the extended minimum at linear geometries that enables further non-adiabatic coupling and formation of H atoms together with internally excited SH(*X*) products. Non-adiabatic coupling to the 1^1^A′ PES *via* CI-2 and/or to the minimum energy seam with the 1^1^A′′ PES at *θ* = 180° also offers a plausible route to the SH(*X*) ‘super-rotors’ identified when exciting at somewhat shorter wavelengths (149.15 ≥ *λ* ≥ 140.89 nm, Fig. S2 and S3†).

SH(*A*) photofragments are observed following excitation at all *λ* ≤ 149.15 nm. The SH(*A*) fragments are also formed in a wide range of *v*′, *N*′ levels. All these SH(*A*) fragments will predissociate to H + S(^3^P) products within a few nanoseconds of their formation. The dominant SH(*A*, low *v*′, high *N*′) fraction has been rationalised in terms of H_2_S(R) molecules that couple to the 2^1^A′ PES and have sufficient axial recoil energy to exceed the critical *r*_SH2_ value associated with CI-2 before achieving linearity.^50^ These molecules remain on the 2^1^A′ PES and dissociate to yield rotationally excited SH(*A*) products. Some of these SH(*A*) fragments are formed in ‘super-rotor’ levels. The extrapolation of such dynamics offers one route to forming atomic 2H + S(^1^D) fragments at the shorter excitation wavelengths. Analogy with H_2_O^39,67,68^ suggests that the balance of axial and tangential (radial and angular) forces experienced by H_2_S molecules evolving on the 2^1^A′ PES may also allow the separating H atom to ‘orbit’ its rotating SH partner as a centrifugally-bound complex and sample CI-1 and the minimum energy seam with the 1^1^A′′ PES at *θ* = 0°. Both S–H bond lengths are extended at the CI-1 minimum energy geometry (*r*_SH1_ = 1.74 Å, *r*_SH2_ = 2.60 Å, Table 2). This *r*_SH1_ value corresponds to the average bond length of SH(*X*) radicals in their *v*′′ = 8 level (see Table S4†). The non-adiabatic coupling *via* CI-1 is thus a plausible contributor to the yield of vibrationally excited SH(*X*) products. As Fig. 9 shows, the non-adiabatic coupling at CI-1 can also offer an intramolecular H atom transfer route to H_2_ + S(^1^D) products.

The S(^1^D) products were only observed following excitation at *λ* ≤ 146.50 nm. Though the foregoing discussion implies that the H_2_S(R) molecules couple to the 2^1^A′ PES at longer excitation wavelengths, the topography of the PES shows a modest energy barrier in the path connecting the vertical region to the S(^1^D) + H_2_ asymptote. Thus, it is logical that S–H bond extension (and fission) dominates, and that S(^1^D) atoms only appear when exciting at somewhat higher energies (shorter wavelengths). The topography of the relevant region of the 2^1^A′ PES favours formation of vibrationally excited H_2_ products, with modest rotational excitation. Thus, the *v*′′, *J*′′ population distributions in the H_2_ partners to the imaged S(^1^D) products observed following excitation to different various H_2_S(R) resonances at *λ* ≤ 146.50 nm can be rationalised as a sum of two components, both of which require initial non-adiabatic coupling to the 2^1^A′ (or 3^1^A′) continuum. The major component, yielding products with high *v*′′ and low *J*′′, involves a concerted elongation of both S–H bonds and some concomitant reduction in *θ* after coupling onto the 2^1^A′ PES, while the minor low *v*′′, high *J*′′ component is attributed to dissociations that begin with extension of one S–H bond, orbiting of the departing H about the SH partner and subsequent H atom transfer at CI-1 and/or at the seam of degeneracy with the 1^1^A′′ PES at *θ* = 0°. Extrapolation of both dynamics could provide other minor routes to atomic 2H + S(^1^D) fragments at the short excitation wavelengths.

The S(^1^D) and, particularly, the S(^1^S) data require that we introduce one further complication. The 2^1^A′ and 3^1^A′ PESs show a conical intersection in the vertical region (CI-6, Fig. S11†), but the present experiments are largely silent regarding to which of these ^1^A′ states any given H_2_S(R) state might couple. However, the 3^1^A′ potential plotted in Jacobi coordinates (Fig. S18†) shows a significantly larger energy barrier from the vertical region to S–H bond fission than to S(^1^D) elimination. Thus, it is tempting to suggest that the detection of S(^1^D) products is a signifier of non-adiabatic coupling to the 3^1^A′ (as well as the 2^1^A′) PES. Such a conclusion would also be consistent with the rationale offered recently for the observed energy disposal in the S(^1^S) + H_2_ products,^58^ which shows parallels with that associated with the major contributor to the S(^1^D) + H_2_ product channel. As Fig. S19† shows, the 3^1^A′ and 4^1^A′ PESs become near degenerate upon stretching both S–H bonds and reducing *θ*, thereby offering a route for flux propagating on the 3^1^A′ PES to couple to the 4^1^A′ PES and dissociate to S(^1^S) + H_2_ products. Again, the dynamics favour vibrational excitation of the H_2_ products, but energy conservation dictates that the distribution will span a smaller range of *v*′′ levels than in the S(^1^D) + H_2_ product channel.

This discussion has focused on the wavelength-dependent photofragmentation dynamics, particularly the exclusive H + SH(*X*) product formation at *λ* > 150 nm, and the progressive appearance of H + SH(*A*), S(^1^D) + H_2_ and S(^1^S) + H_2_ products as the excitation energy is increased. Most of the resonances excited in the present work are assigned as having ^1^B_1_ symmetry.^43^ (Our proposed reassignment of the *λ* ∼ 154.5 nm band merely compounds this statement). In all cases, excitation of these resonances yields some H + SH(*X*) products, with preferential perpendicular recoil anisotropies. The SH(*X*)/SH(*A*) yield generally declines with decreasing wavelength, though not monotonically. The lowest SH(*X*)/SH(*A*) yields are observed when exciting at *λ* = 129.95 nm, 122.95 nm and, previously, at *λ* = 121.6 nm.^49,50^ The reported symmetries of the states populated at these wavelengths are, respectively, ^1^B_2_, ^1^A_1_ and ^1^A_1_ (*i.e.* all ^1^A′ in *C*_s_),^43^ hinting that the product energy disposals could offer a clue to the R state symmetries.

## Conclusions

**Fig. 11:**
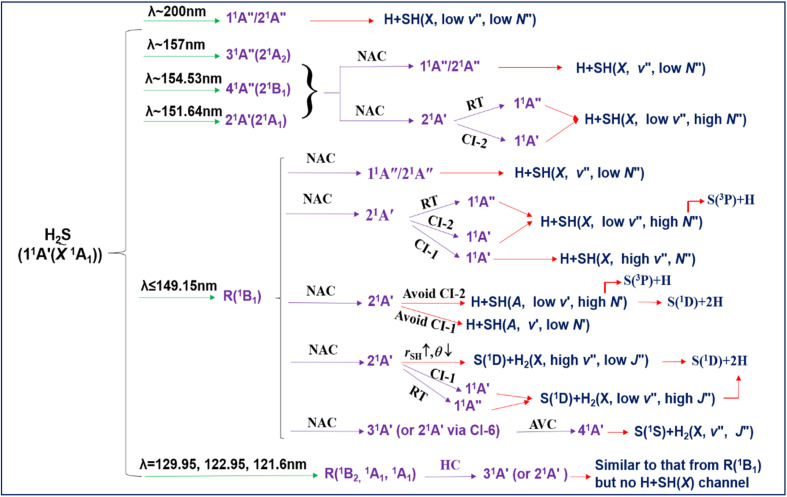
The schematic overview of the wavelength and vibronic state dependent photofragmentation dynamics of H_2_S. R: Rydberg state; NAC: non-adiabatic coupling; RT: Renner–Teller coupling; CI: passage through conical intersection (unless specified otherwise); AVC: avoided crossing; HC: homogeneous (vibronic) coupling.

We have presented a comprehensive investigation of the wavelength and state dependent photodissociation dynamics of H_2_S, which have been summarized in Fig. 11. Excitation to the long wavelength 2^1^A′′/1^1^A′′ continuum (*λ* ∼ 200 nm) yields H + SH(*X*, *v*′′, low *N*′′) products. The first Rydberg (R) state resonances appear in the *λ* ∼ 150–160 nm region. These, too, dissociate to H + SH(*X*) products, but the latter are formed in a wide range of *v*′′, *N*′′ levels, stretching all the way up to a small yield of the triple dissociation products S(^3^P) + 2H. Dissociation in this case is deduced to involve initial non-adiabatic coupling of the R state population to the bottom of the 2^1^A′ continuum. The available energy is insufficient to allow adiabatic dissociation (to H + SH(*A*) products) to compete with rival non-adiabatic pathways from the 2^1^A′ state to the 1^1^A′′ and/or 1^1^A′ states and dissociation to H + SH(*X*) products. The parent geometry changes associated with the latter non-adiabatic couplings map through as rovibrational excitation of the SH(*X*) products.

The H + SH(*A*) products are observed following excitation at all *λ* < 150 nm. The SH(*A*) products show extensive rotational extension which, again, can be rationalised by considering the topography of the 2^1^A′ PES. The longest wavelengths at which S(^1^D) and S(^1^S) atoms detected are, respectively, *λ* = 146.50 nm and 143.00 nm. Both are deduced to be minor channels throughout the wavelength range studied. The internal energy distribution within the H_2_ partner fragments in both cases appears to partition into two components: a larger yield of high *v*′′, low *J*′′ products and a lesser yield with low *v*′′ and high *J*′′. The former is attributed to dissociation after non-adiabatic coupling to a ^1^A′ PES (arguments are presented favouring the 3^1^A′ PES) and an essentially barrierless parent distortion wherein both S–H bonds extend in tandem with a reduction in the interbond angle *θ*. The latter fraction is attributed to dissociations proceeding *via* non-adiabatic coupling on the 2^1^A′ PES, extension of one S–H bond, orbiting of the departing H about the SH partner, and subsequent H atom transfer at linear H⋯HS geometries (*θ* = 0°).

Three of the R states excited at shorter wavelengths and assigned as having ^1^B_2_ or ^1^A_1_ (*i.e.*^1^A′) symmetry are found to yield no H + SH(*X*) products. This is to be expected, if the dominant decay route for these states is homogeneous coupling to the 3^1^A′/2^1^A′ continuum and subsequent S–H bond extension with sufficient axial kinetic energy to evade CI-2 and remain on (and dissociate on) the 2^1^A′ PES. The R states of ^1^B_1_ symmetry (or of ^1^A_2_ symmetry if optically accessible), in contrast, will always have one or two low *J*′ rotational levels that cannot couple to a continuum of ^1^A′ symmetry. The fraction of the total population in such states will be much larger when using jet-cooled (as in the present experiments) rather than thermal samples. Such levels can only dissociate by coupling to the ^1^A′′ continuum and dissociating to ground state products. Thus, the SH(*X*)/SH(*A*) product yields measured under jet-cooled conditions are proposed as a means of distinguishing R states of ^1^A′ and ^1^A′′ symmetry.

This study offers the most complete picture yet available of the VUV photofragmentation behaviour of H_2_S. From the perspective of astrochemical modelling, all of these fragmentation channels should have a role in the evolution of the interstellar clouds. For instance, the SH(*X*)/H_2_S abundance ratios returned by the astronomical observations imply that the SH(*X*) radical is depleted relative to that predicted by commonly-used astrochemical models. Our results indicate that most H_2_S molecules exposed to the general ISRF undergo eventual triple fragmentation to 2H + S(^3^P) products. Only about one quarter of H_2_S photoexcitation events induced by the general ISRF yield SH(*X*) products, which implies a need for a revision of relevant astrochemical models.^26^ In addition, the S(^1^D) and S(^1^S) elimination channels are deduced to have small quantum yields, which provide not merely formation routes for the S atoms in the coma of comets like C/1995 O1 (ref. 69) and C/2014 Q2,^70^ but also sources of vibrationally excited H_2_ molecules.^71^ Though the vibrational excited interstellar H_2_ mainly comes from the shock waves and far-ultraviolet fluorescence, the photochemical processes observed in this work may contribute to the highly ro-vibrationally excited interstellar H_2_.^58^ All these findings should be recognised in refined recommendations for future astrochemical modelling. It is also very much hoped that the comprehensive data reported in this work will serve to inspire further molecular dynamics studies of the nuclear motions on, and the non-adiabatic couplings between, the photochemically accessible excited states of H_2_S so that this molecule can represent as a textbook example of a small polyatomic molecule with an unusually fully described photochemistry.

## Author contributions

K. J. Y. and X. M. Y. supervised the research. K. J. Y. conceived the research. M. N. R. A. and K. J. Y. designed the experiments. Y. R. Z., Z. J. L., and Y. C. performed the experiments. J. Y. Y., W. Q. Z., G. R. W., and X. M. Y. operated the FEL facility. Y. R. Z., Z. J. L., S. W. C., C. S. H., H. B. D., and M. N. R. A. analysed the data. J. J. C., A. F., X. X. H., and D. Q. X. performed the electronic structure calculations. Y. R. Z., X. X. H., K. J. Y., and M. N. R. A. wrote the manuscript. All authors discussed the results and commented on the manuscript.

## Data availability

All associated experimental data can be found in ESI.†

## Conflicts of interest

There are no conflicts to declare.

## Supplementary Material

SC-014-D2SC06988A-s001
