# Supplementary material for: The vibronic state dependent predissociation of H2S: determination of all fragmentation processes
Source: Chem Sci. 2023 Feb 14;14(10):2501–17. doi: 10.1039/d2sc06988a (PMC9993885; doi:10.1039/d2sc06988a)
Supplement: SC-014-D2SC06988A-s001 [file SC-014-D2SC06988A-s001.pdf]

Electronic Supplementary Material (ESI) for Chemical Science.
This journal is © The Royal Society of Chemistry 2023

Electronic Supplementary information (ESI)

The Vibronic State Dependent Predissociation of H_2S: Determination of All Fragmentation Processes

Yarui Zhao,‡[a,b] Junjie Chen,‡[c] Zijie Luo,‡[b] Yao Chang,[b] Jiayue Yang,[b] Weiqing Zhang,[b] Guorong Wu,[b] Stuart W. Crane,[d] Christopher S. Hansen,[e] Hongbin Ding,[a] Feng An,[c] Xixi Hu,*[f,g] Daiqian Xie,[c,g] Michael N.R. Ashfold,*[d] Kaijun Yuan,*[b,g] Xueming Yang[b,g,h]

[a] *School of Physics, Key Laboratory of Materials Modification by Laser, Ion and Electron Beams, Chinese Ministry of Education, Dalian University of Technology, Dalian, 116024, China*

[b] *State Key Laboratory of Molecular Reaction Dynamics and Dalian Coherent Light Source, Dalian Institute of Chemical Physics, Chinese Academy of Sciences, Dalian 116023, China.*

[c] *Institute of Theoretical and Computational Chemistry, Key Laboratory of Mesoscopic Chemistry, School of Chemistry and Chemical Engineering, Nanjing University, Nanjing 210023, China*

[d] *School of Chemistry, University of Bristol, Bristol, BS8 1TS, UK*

[e] *School of Chemistry, University of New South Wales, Sydney, NSW 2052, Australia*

[f] *Kuang Yaming Honors School, Institute for Brain Sciences, Jiangsu Key Laboratory of Vehicle Emissions Control, Center of Modern Analysis, Nanjing University, Nanjing 210023, China*

[g] *Hefei National Laboratory, Hefei 230088, China*

[h] *Department of Chemistry, Southern University of Science and Technology, Shenzhen 518055, China*

Authors for correspondence:

Xixi Hu, Michael N.R. Ashfold, Kaijun Yuan

E-mail addresses: xxhu@nju.edu.cn, mike.ashfold@bristol.ac.uk, kjyuan@dicp.ac.cn.

‡ These authors contributed equally to this work.

Experimental methods

The H atom product translational energy distributions were recorded using a tuneable vacuum ultraviolet (VUV) free-electron laser (FEL) along with the H-atom Rydberg tagging time-of-flight (HRTOF) probe technique, performed using the recently constructed end-station for molecular photochemistry around the VUV FEL beam line at the Dalian Coherent Light Source (DCLS).[1, 2] The VUV FEL operates in the high gain harmonic generation mode, in which the seed laser (λ ~ 240–360 nm) generated from a Ti: sapphire laser is injected to interact with the electron beam in the modulator. The electron beam is generated from a photocathode RF gun and accelerated to a beam energy of ~300 MeV by 7 S-band accelerator structures, with a bunch charge of 500 pC. The micro-bunched beam is then sent through the radiator, which is tuned to the *n*th harmonic of the seed wavelength, and coherent FEL radiation with wavelength λ/*n* is emitted. The optimization of the linear accelerator yields a high-quality light beam with an emittance of ~1.5 mm mrad, an energy spread of ~1‰, a pulse duration of ~1.5 ps and maximum pulse energies >100 μJ pulse^{-1}. In the present study, the VUV-FEL operated at 10 Hz, and a typical spectral bandwidth is ~50 cm^{-1}.

In the HRTOF detection method, the H atom products are promoted from the ground state to a high n Rydberg state via a two-step excitation. Step one involves resonant excitation from the n = 1 to n = 2 state at the Lyman-α wavelength (λ = 121.6 nm), while step two uses UV laser excitation at λ ~365 nm to further excite the H atom from the n = 2 state to a high-n (n = 30–80) Rydberg state lying slightly below the ionization threshold. The coherent 121.6 nm radiation was generated by difference four-wave mixing (DFWM) involving two 212.556 nm photons and one 845.384 nm photon overlapped in a stainless-steel cell filled with a 3:1 ratio Ar/Kr gas mixture. The neutral Rydberg-tagged H atom photofragments flew a distance *d* ~280 mm before reaching a grounded mesh mounted close in front of Z-stack rotatable micro-channel plate (MCP) detector, where they were immediately field ionized by the ~ 2000 V cm^{-1} electric field. The signal detected by the MCP was then amplified by a fast pre-amplifier and counted by a multichannel scaler. The recorded TOF data were converted to the corresponding H atom kinetic energy distributions based on momentum conservation, as shown in the main text. The sample beam was generated by expanding a mixture of 1% H_2S and Ar at a stagnation pressure of 760 Torr through a 0.5 mm-diameter pulsed nozzle. The molecular beam crossed the VUV-FEL beam at right angles.

The S(^{1}D) / S(^{1}S) atom product translational energy distributions were recorded using the VUV FEL pump time-sliced velocity map imaging (TSVMI) probe technique[3, 4], which

involves a molecular beam, photolysis and probe lasers, and the detection system. The pulsed supersonic beam was again generated by expanding a mixture of 1% H_2S and Ar at a stagnation pressure of 760 Torr into the source chamber where it was skimmed before entering the ion optics assembly (IOA, 23-plate ion optics). The beam passed through a 2 mm hole in the first electrode and propagated along the centre axis of the IOA towards the centre of the front face of the detector. The molecular beam was intersected at 90 ° angles by the counter-propagating photolysis and probe laser beams between the second and the third plates of the IOA. The photolysis photons were again provided by the FEL, and the polarization of the VUV-FEL pulse was fixed in the horizontal plane and thus parallel to the front face of the detector. The $S(^1D)$ photoproducts were probed by one-photon excitation at $\lambda = 130.092$ nm, which populates the autoionizing $3p^3(^2D^o)5s$; $^1D_2{}^o$ level, while $S(^1S)$ fragments were resonantly ionized at $\lambda = 136.13$ nm via the $S^*[3s^23p^3(^2P^o)5s^1(^1P^o)] \leftarrow S[3s^23p^4(^1S_0)]$ transition. These probe photons were generated by the DFWM, involving two 212.556 nm photons together with one 580.654 nm or 486.133 nm photon from laser pulses that were overlapped in a Kr-containing gas cell (for $S(^1D)$ and $S(^1S)$ detection, respectively). The resulting S^+ ions were then accelerated through the remainder of the IOA and passed through a 740 mm long field-free region before impacting on a 75 mm-diameter chevron double MCP detector coupled with a P47 phosphor screen. Transient images on the phosphor screen were recorded by a charge-coupled device (CCD) camera. Converting the radius of any given feature in the measured images to the corresponding S atom velocity relied on calibration factors derived from imaging O^+ ions from the one-colour multiphoton excitation of O_2 at $\lambda = 225.00$ nm.

Computation methods

Electronic Structure calculations for the five lowest-lying states of both $^1A'$ and $^1A''$ symmetry was performed at the internally contracted multi-reference configuration interaction corrected with Davidson correction (ic-MRCI+Q) level. To obtain appropriate reference wavefunctions for the MRCI calculation, a prior state-averaged completed active space self-consistent field (SA-CASSCF) calculation containing 18 A′ states and 18 A″ states was implemented. To provide a precise description of the highly excited Rydberg states, an exceptionally large active space (8*e*,13*o*) composed of nine a′ and four a″ active orbitals in C_s symmetry was applied. In addition, quite large basis sets were used for this system. The basis set for the S atom was a correlation-consistent, polarized, valence, quadruple zeta (cc-pVQZ) basis extended by six diffuse s functions, six diffuse p functions and eight diffuse d functions. For the H atoms, a cc-pVQZ basis set extended by two diffuse s functions and two diffuse p

functions was used. All the aforementioned *ab initio* calculations were performed using the MOLPRO 2015.1 package.

Potential energy surfaces were fitted using the Gaussian Process Regression (GPR) method based on >700 points covering both the interaction region and the two dissociation channels. The permutation symmetry of the two identical H atoms in H_2S was accommodated using permutation invariant polynomials (PIPs).

Oscillator strengths for transitions from the $1^1A'$ ground state to the first four excited states of A′ symmetry and the first five excited states of A″ symmetry were obtained by equation-of-motion coupled cluster single and double excitation (EOM-CCSD) calculations at the ground state equilibrium geometry (θ_{HSH} = 92.2 °, r_{SH1} = r_{SH2} = 1.34 Å). Inspection of the results listed in Tables 1 and S1 confirms that the discrepancies between the VEEs calculated using the EOM-CCSD and MRCI methods are reassuringly small (≤ 0.1 eV in all cases).

Electronic State	VEE / eV	*f*
$2^1A'(2^1A_1)$	8.24	2.63×10^{-4}
$3^1A'(1^1B_2)$	8.76	0.037
$4^1A'(3^1A_1)$	8.85	0.042
$5^1A'(4^1A_1)$	9.30	0.079
$1^1A''(1^1A_2)$	6.20	0.000
$2^1A''(1^1B_1)$	6.33	0.060
$3^1A''(2^1A_2)$	7.92	0.000
$4^1A''(2^1B_1)$	8.02	3.56×10^{-3}
$5^1A''(3^1B_1)$	8.38	0.069

Table S1. The vertical excitation energies (VEEs) and oscillator strengths, *f*, for excitation from the ground state equilibrium geometry to the first four excited states of $^1A'$ states and the five lowest $^1A''$ electronic states obtained from the EOM-CCSD calculations.

Dissociation channel	Calculated dissociation Energy / eV	Experimental value / eV
SH($X^2\Pi$) + H(^{2}S)	3.87	3.90
SH($A^2\Sigma^+$) + H(^{2}S)	7.68	7.70
SH($B^2\Sigma^-$) + H(^{2}S)	11.23	11.29
SH($C^2\Delta$) + H(^{2}S)	11.80	11.84
S(1^1D) + H_2($X^1\Sigma_g^+$)	4.10	4.20
S(^{1}S) + H_2($X^1\Sigma_g^+$)	5.78	5.80
S(2^1D) + H_2($X^1\Sigma_g^+$)	11.72	11.68
S(^{3}P) + 2H(^{2}S)	7.48	7.53
S(^{1}D) + 2H(^{2}S)	8.61	8.68
S(^{1}S) + 2H(^{2}S)	10.26	10.28

Table S2. The calculated dissociation energies for fragmentation channels of H_2S relevant to the present study, after correction for differences in zero-point energy, along with the best-estimate experimental (D_0) values (eqs. (1)–(6) in the main paper), plus term values for the *B* and *C* excited states of SH.

electronic state	θ_{HSH} / °	r_{SH1} / Å	r_{SH2} / Å	E / eV	dissociation products
$2^1A'$	66.89	1.51	1.51	8.79	$H_2(X^1\Sigma_g^+)$ + $S(^1D)$
	104.06	1.38	1.38	8.30	H + $SH(A^2\Sigma^+)$
$3^1A'$	67.11	1.56	1.56	9.20	$H_2(X^1\Sigma_g^+)$ + $S(^1D)$
$4^1A'$	40.35	1.63	1.63	10.31	$H_2(X^1\Sigma_g^+)$ + $S(^1S)$
$1^1A''$	42.93	1.60	1.60	6.11	$H_2(X^1\Sigma_g^+)$ + $S(^1D)$
$2^1A''$	37.16	2.11	2.11	7.83	$H_2(X^1\Sigma_g^+)$ + $S(^1D)$

Table S3. The geometries and relative energies (without zero-point correction) of the optimized transition state along the H + SH and/or S + H_2 dissociation pathways for the $2^1A'$, $3^1A'$, $4^1A'$, $1^1A''$ and $2^1A''$ electronic states of H_2S.

v''	Energy / cm^{-1}	$\bar{r}_{SH}$ / Å
0	0	1.36
1	2611.7	1.39
2	5112.0	1.43
3	7499.7	1.47
4	9773.8	1.51
5	11932.8	1.56
6	13975.3	1.61
7	15899.9	1.66
8	17704.6	1.71
9	19387.7	1.78
10	20946.8	1.85
11	22379.6	1.92
12	23683.1	2.01
13	24854.1	2.11

Table S4. The calculated energies and average bond lengths of SH(*X*) radicals in different vibrational v'' states.

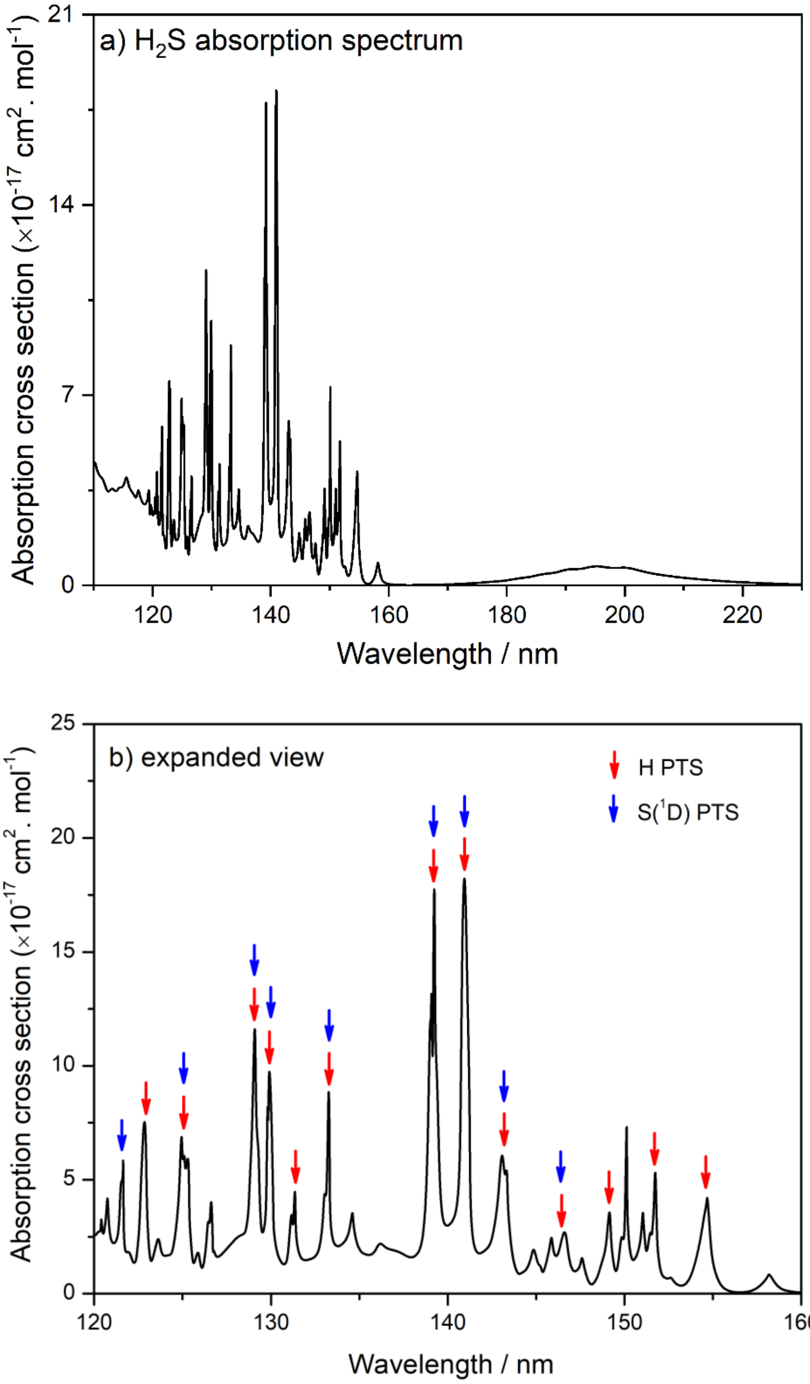

Fig. S1. H_2S absorption cross-section versus wavelength. The expanded view in the lower panel indicates the resonances on which H atom PTS (red arrow) and $S(^1D)$ atom PTS (blue arrow) studies were conducted.

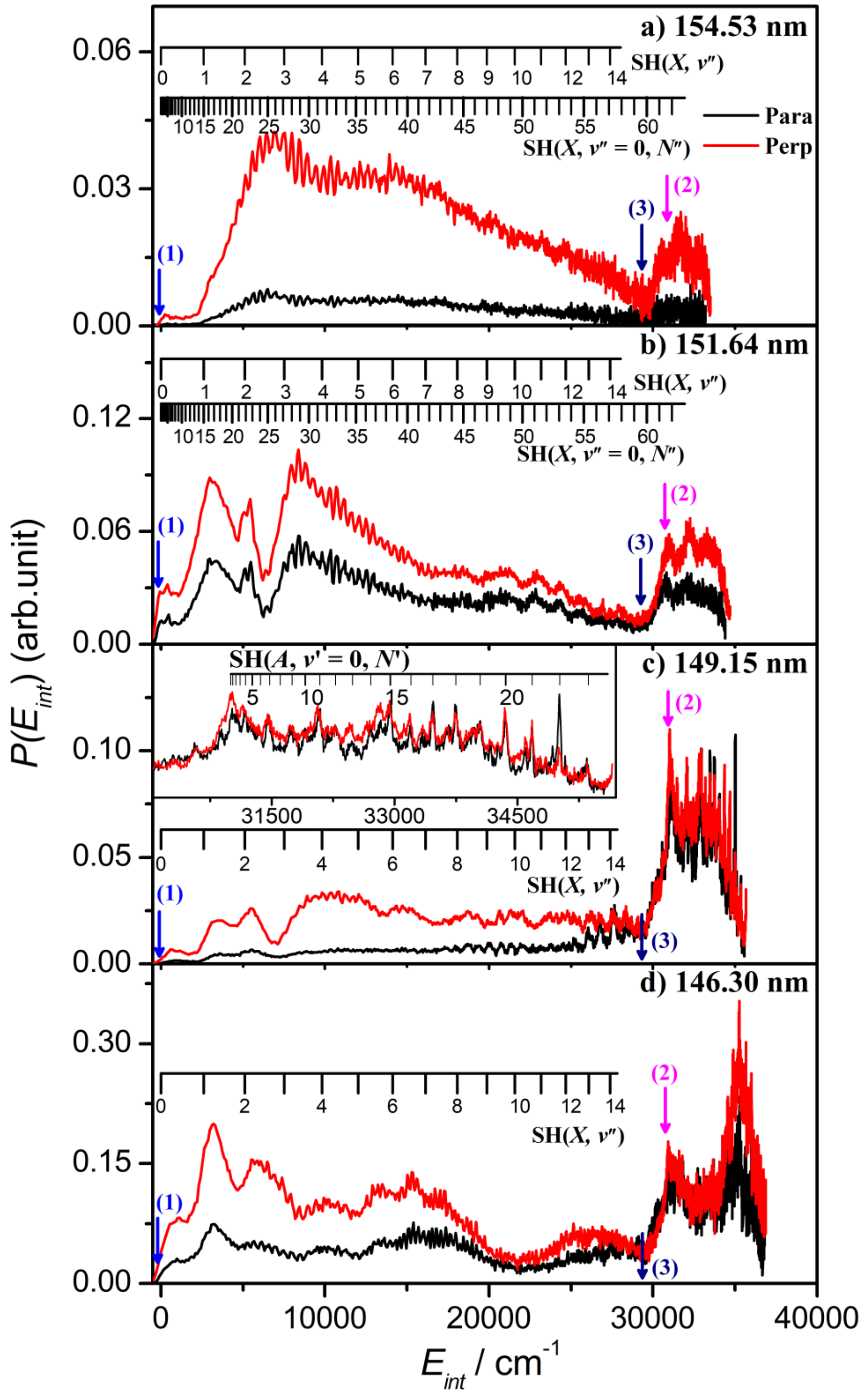

Fig. S2. The $P(E_{\mathrm{int}})$ distributions derived from H atom TOF spectra following photodissociation of H_2S at λ = (a) 154.53 nm, (b) 151.64 nm, (c) 149.15 nm and (d) 146.30 nm, with the detection axis aligned parallel (black) and perpendicular (red) to the $\boldsymbol{\varepsilon}$ vector of the photolysis laser radiation. The inset in (c) shows an expanded view of the high energy part of the $P(E_{\mathrm{int}})$ spectra. The superposed combs show the E_{int} values associated with formation of H atoms along with selected rovibrational levels of the primary SH(X) and SH(A) fragments. The threshold energies associated with channels (1) – (3) are shown by vertical blue, magenta and navy arrows.

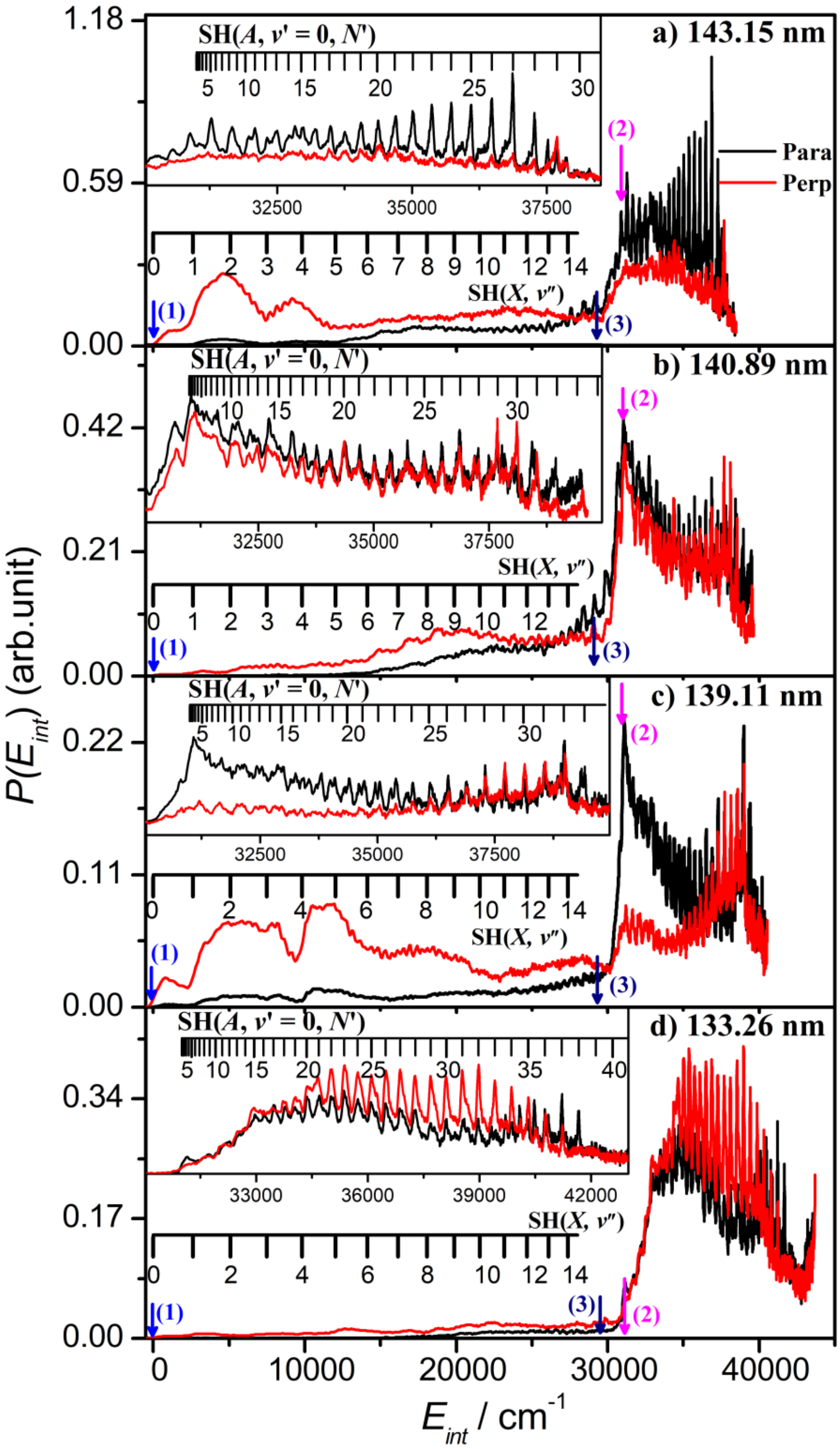

Fig. S3. The $P(E_{\mathrm{int}})$ distributions derived from H atom TOF spectra following photodissociation of H_2S at λ = (a) 143.15 nm, (b) 140.89 nm, (c) 139.11 nm and (d) 133.26 nm, with the detection axis aligned parallel (black) and perpendicular (red) to the **ε** vector of the photolysis laser radiation. The insets show expanded views of the high energy part of the respective $P(E_{\mathrm{int}})$ spectra. The superposed combs show the E_{int} values associated with formation of H atoms together with selected rovibrational levels of the primary SH(X) and SH(A) fragments. The threshold energies associated with channels (1) – (3) are shown by vertical blue, magenta and navy arrows.

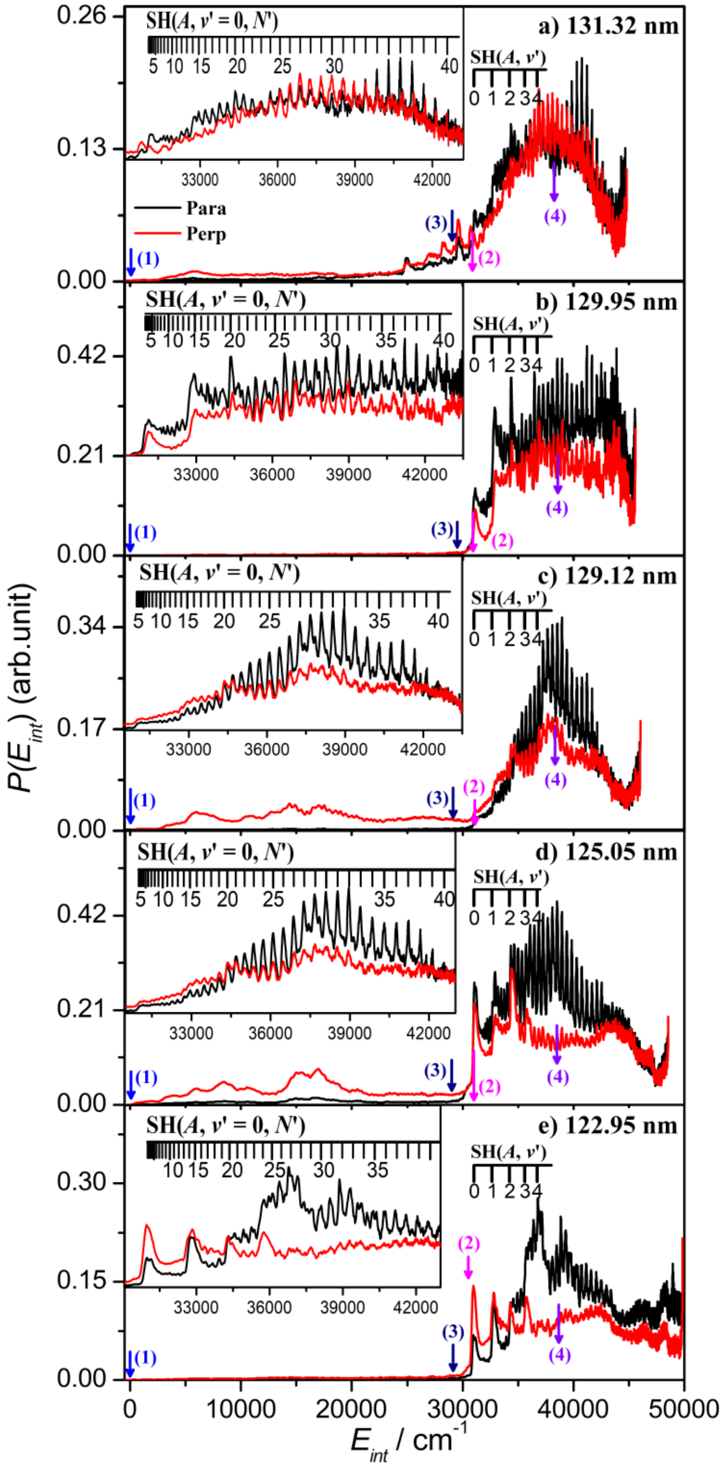

Fig. S4. The $P(E_{\mathrm{int}})$ distributions derived from H atom TOF spectra following photodissociation of H_2S at λ = (a) 131.32 nm, (b) 129.95 nm, (c) 129.12 nm (d) 125.05 nm and (e) 122.95 nm, with the detection axis aligned parallel (black) and perpendicular (red) to the **ε** vector of the photolysis laser radiation. The insets show expanded views of the high energy part of the respective $P(E_{\mathrm{int}})$ spectra. The superposed combs show the E_{int} values associated with formation of H atoms together with selected rovibrational levels of the primary SH(X) and SH(A) fragments; fine structure at E_{int} > 38480 cm^{-1} is attributable to population of SH(A) 'super-rotor' levels. The threshold energies associated with channels (1) – (4) are shown by vertical blue, magenta, navy and violet arrows.

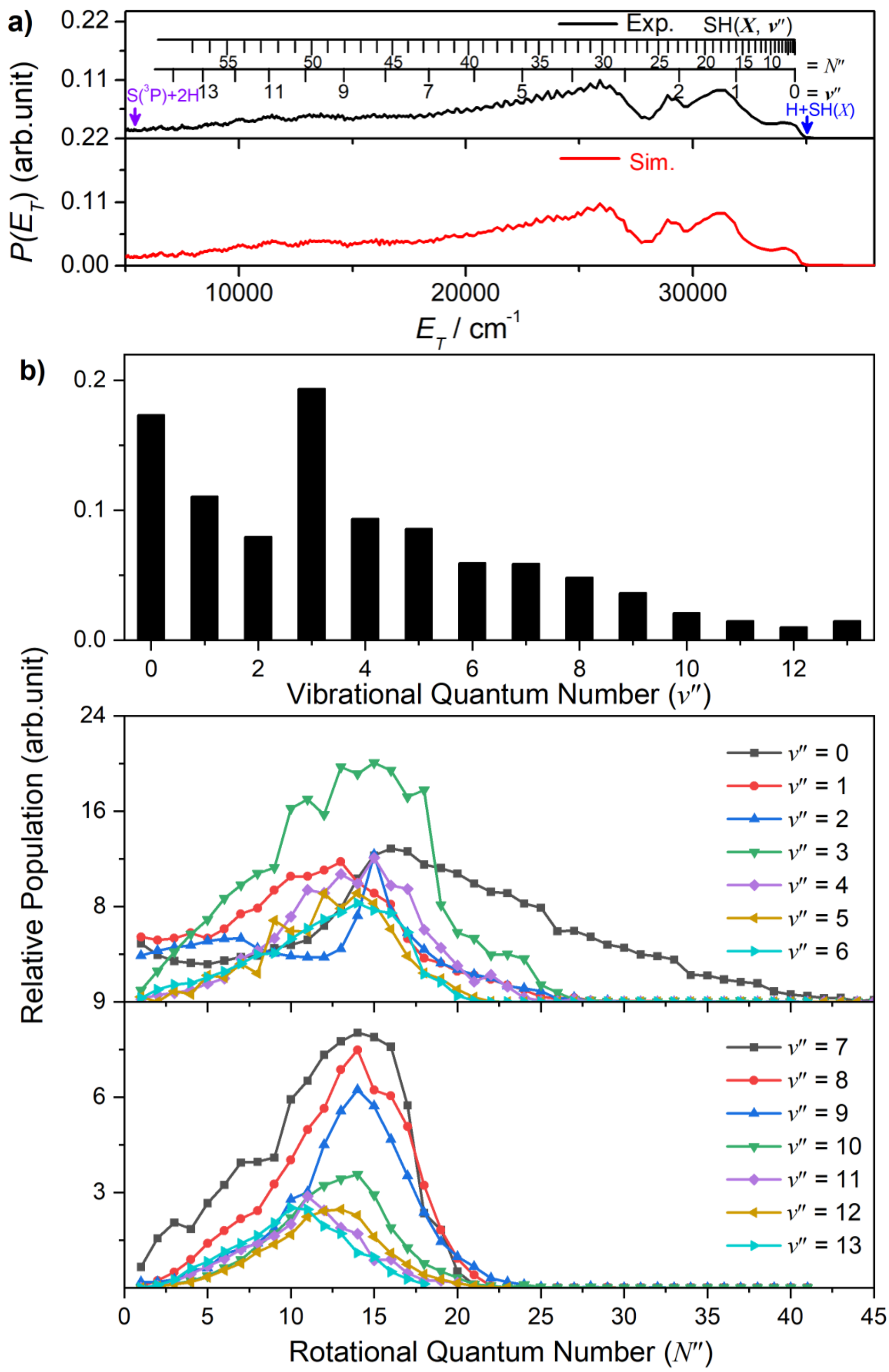

Fig. S5. (a) The best-fit simulation (red) of the experimental (black) $P(E_T)$ spectrum derived from the H atom TOF spectrum obtained following photodissociation of H_2S at $\lambda = 151.64$ nm, measured along a detection axis aligned at $\theta = 54.7°$ to the **ε** vector of the photolysis laser radiation, together with (b) the SH(X) product vibrational and rotational level population distributions derived therefrom.

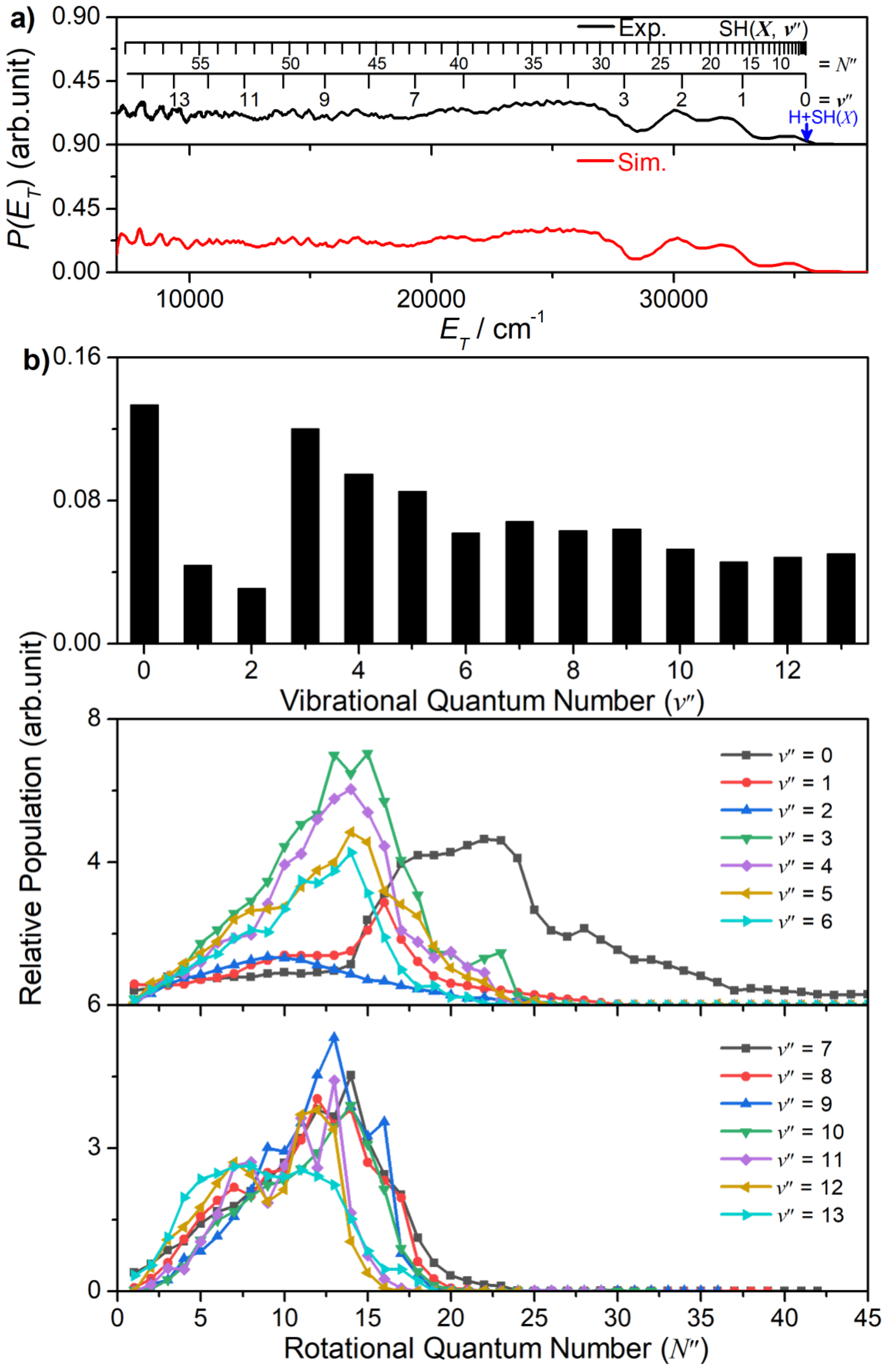

Fig. S6. (a) The best-fit simulation (red) of the experimental (black) $P(E_T)$ spectrum derived from the H atom TOF spectrum obtained following photodissociation of H_2S at λ = 149.15 nm, measured along a detection axis aligned at θ = 54.7° to the **ε** vector of the photolysis laser radiation, together with (b) the SH(X) product vibrational and rotational level population distributions derived therefrom.

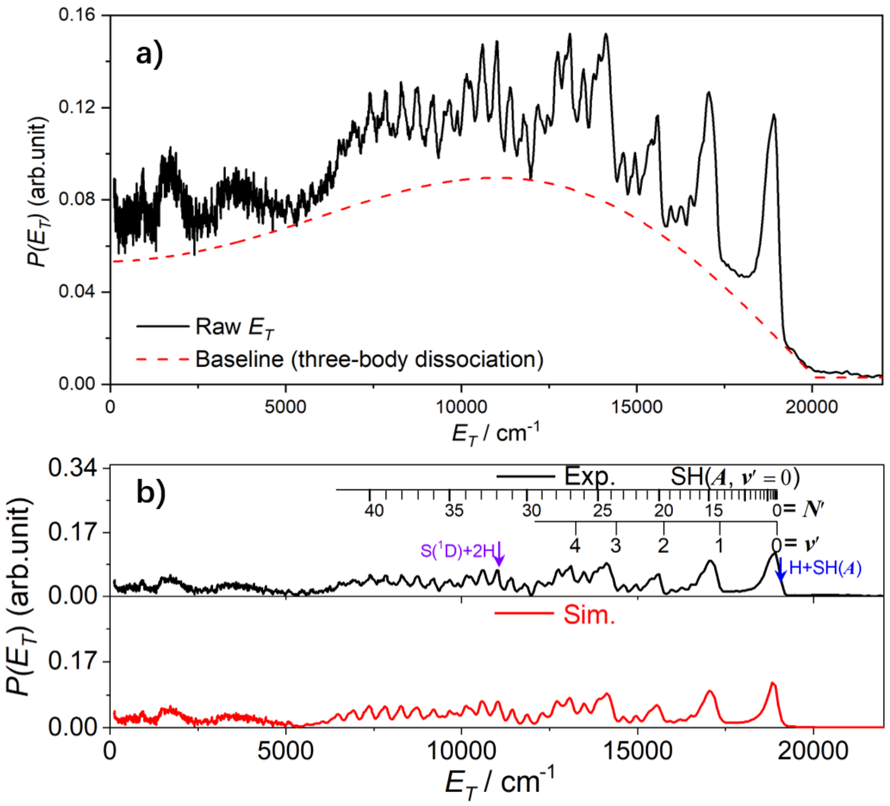

Fig. S7. (a) The $P(E_T)$ spectrum derived from the H atom TOF spectrum following photodissociation of H_2S at λ = 122.95 nm, measured along the detection axis aligned at θ = 54.7° to the **ε** vector, the red dotted curve illustrates the fraction attributed to the three body dissociation. (b) The best-fit simulation (red) of the post-subtracted $P(E_T)$ spectrum (black).

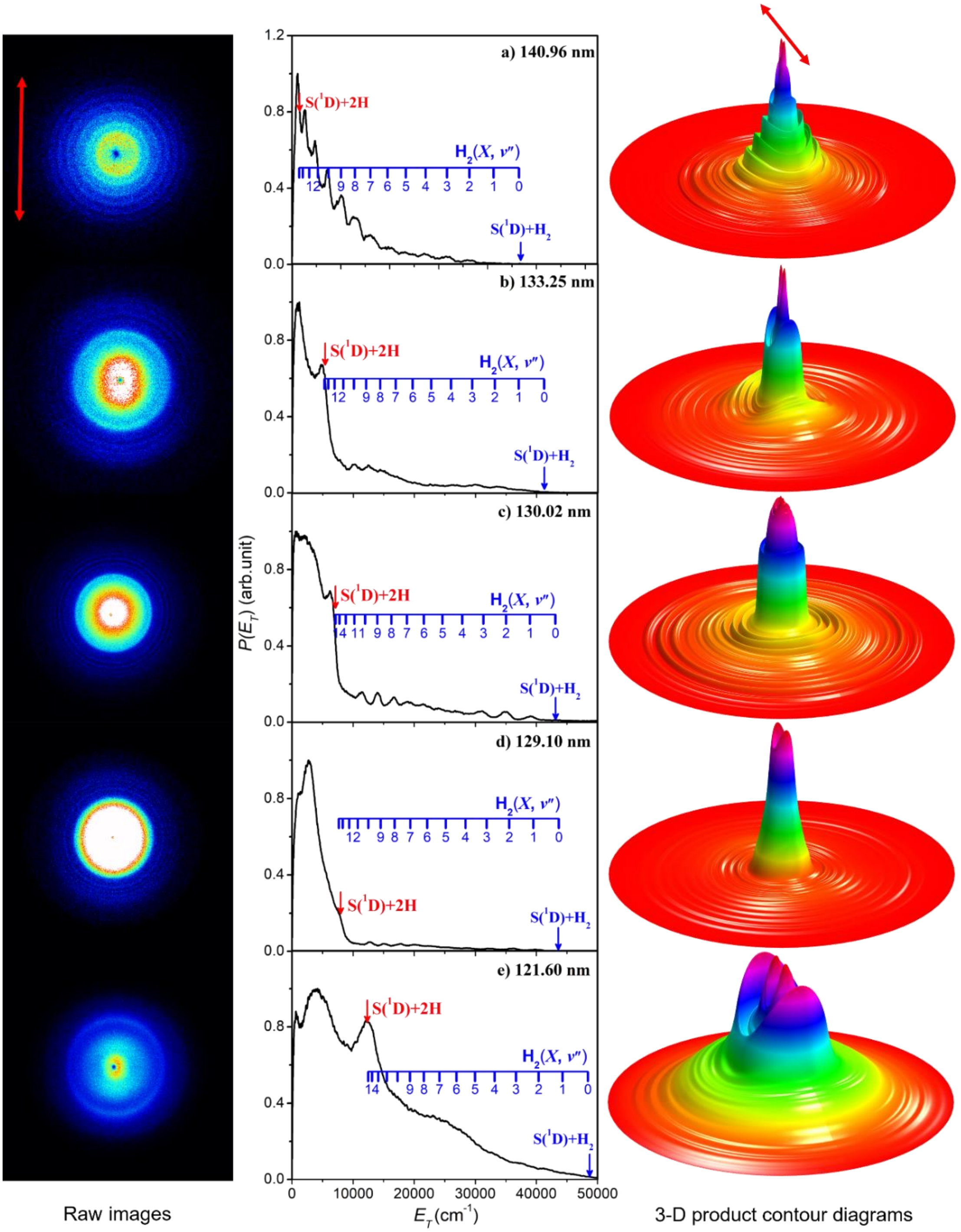

Fig. S8. Time-sliced velocity map images of the S(^{1}D) photofragments from photolysis of H_2S at λ = (a) 140.96 nm, (b) 133.25 nm, (c) 130.02 nm, (d) 129.10 nm and (e) 121.60 nm, with the **ε** vector of the photolysis laser radiation (shown by the double headed red arrow) aligned vertically in the plane of the image. The corresponding $P(\theta, E_T)$ diagrams are shown at the right of each row, with the **ε** vector again indicated by the red arrow on the top diagram. The centre columns show the $P(E_T)$ distributions derived from analysis of these images (assuming H_2 as the partner fragment), with the superposed combs indicating the $H_2(v'')$ states responsible for the evident structure. The navy and red vertical arrows indicate the maximum E_T values associated with, respectively, channels (5) and (4).

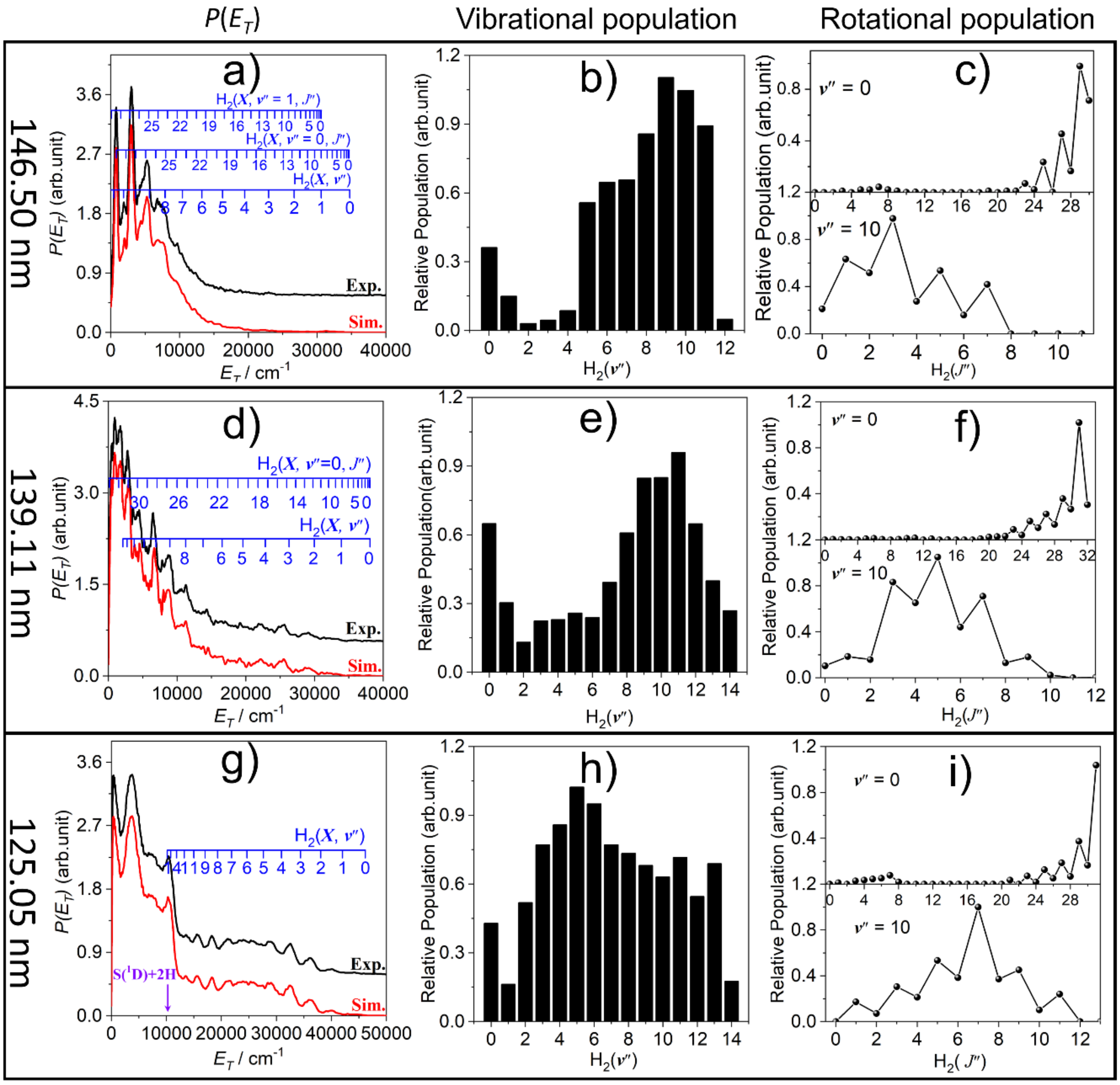

Fig. S9. The $P(E_T)$ distributions derived from time-sliced velocity map images of the $S(^1D)$ photofragments from photolysis of H_2S at λ = (a) 146.50 nm, (d) 139.11 nm and (g) 125.05 nm (black), assuming H_2 as the partner fragment, along with best-fit simulations (red), offset vertically for clarity. The superposed combs show the E_T values associated with H_2 (v'', J''=0), (v''=0, J'') and, in panel (a) only, (v''=1, J'') states that contribute to the evident structure. The H_2 vibrational population distributions derived from the best-fit simulations are shown in panels (b), (e) and (h), respectively, whilst the corresponding rotational level population distributions for the $H_2(v''=0)$ and $H_2(v''=10)$ products formed at each wavelength are shown in panels (c), (f) and (i). The navy vertical arrow in panel (g) indicates the maximum E_T value associated with dissociation channel (4).

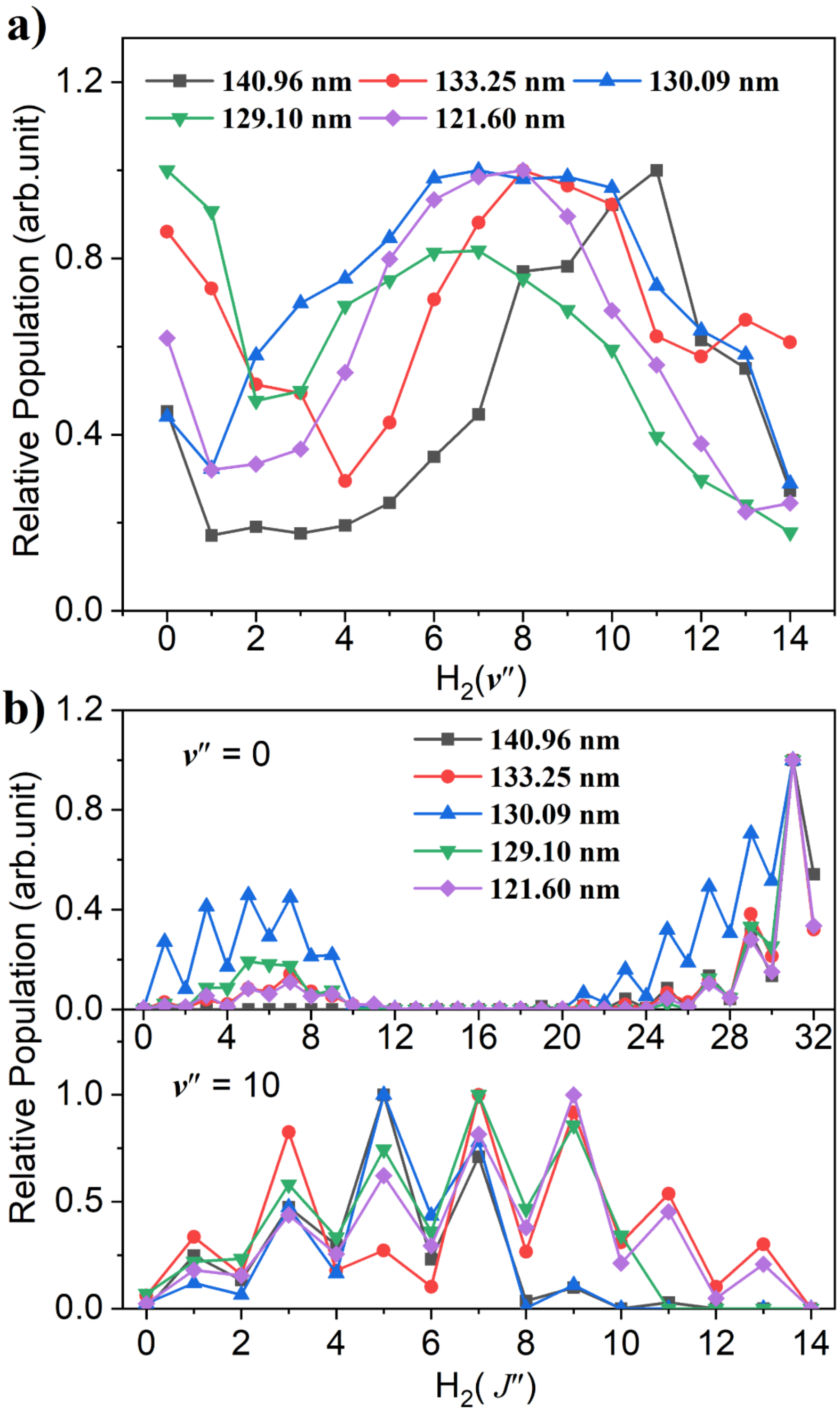

Fig. S10. (a) The vibrational state population distributions of the H_2 products arising via channel (4) in the photolysis of H_2S at λ = 140.96 nm, 133.25 nm, 130.09 nm, 129.10 nm and 121.60 nm derived by simulating the $P(E_T)$ distributions from the time-sliced velocity map images of the $S(^1D)$ photofragments. The corresponding rotational state population distributions of the H_2 products formed in representative low (v''=0) and high (v''=10) vibrational states at each photolysis wavelength are shown in panel (b).

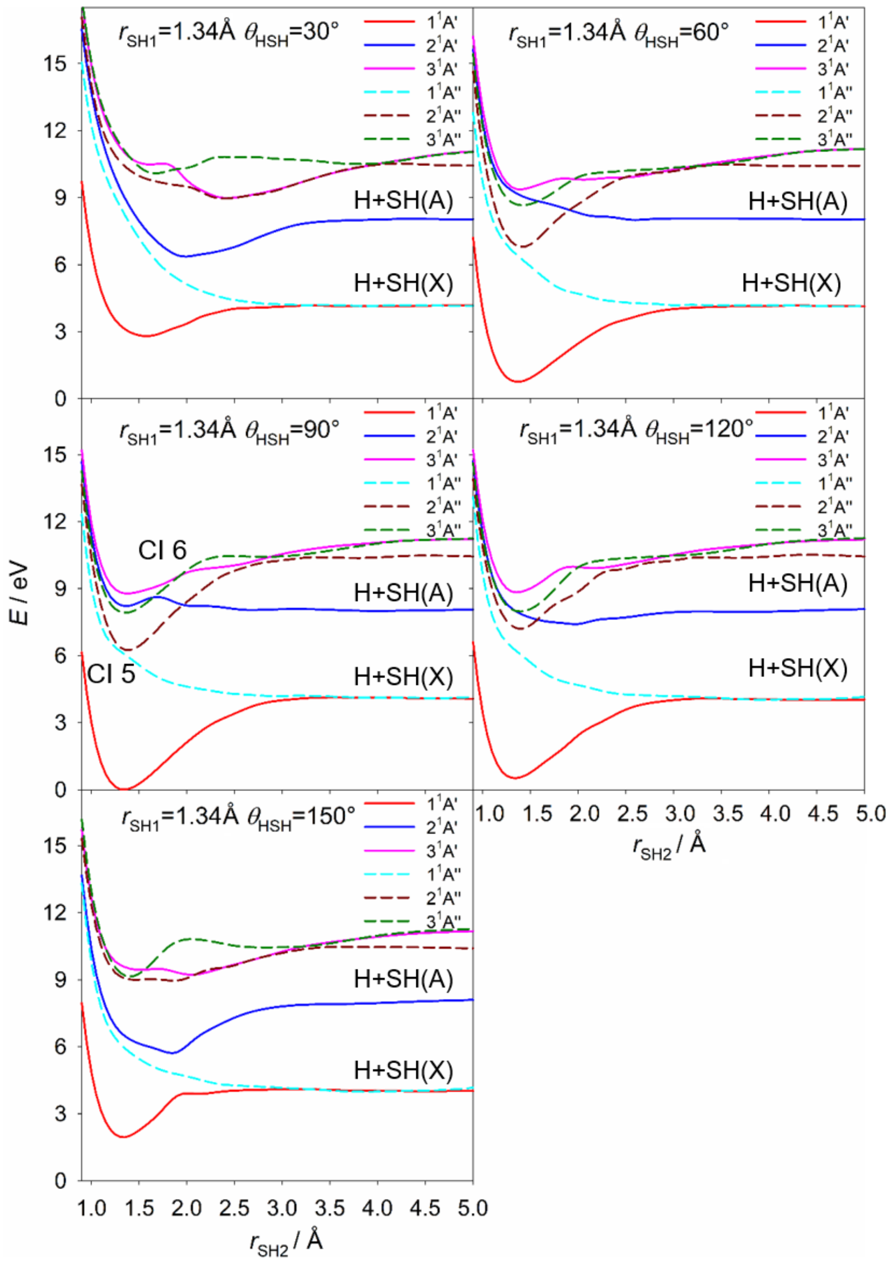

Fig. S11. The Cuts through the calculated PESs for the three lowest energy states of H_2S with each of $^1A'$ and $^1A''$ symmetry, at ∠HSH bond angles θ = 30, 60, 90, 120 and 150°, plotted as a function of one S–H bond length, r_{SH2}, with the other held fixed at its ground state equilibrium value (r_{SH1} = 1.34 Å).

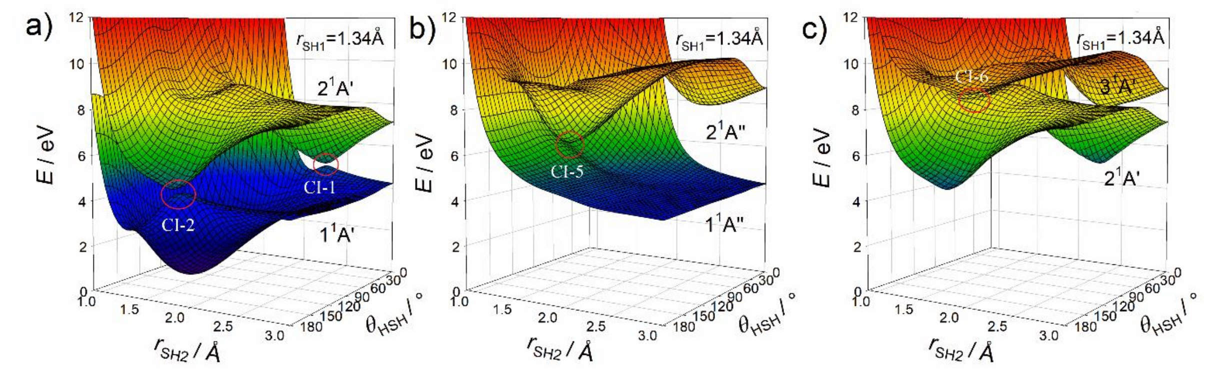

Fig. S12. The 3-D plots showing the topography of selected pairs of potentials (plotted as functions of r_{SH2} and θ, with r_{SH1} fixed at its equilibrium value (1.34 Å)): (a) $1^1A'$ and $2^1A'$ PESs, highlighting CI-1 and CI-2 at linear geometries; (b) $1^1A''$ and $2^1A''$ PESs, showing CI-5, accessible by near-vertical excitation from the (bent) ground state equilibrium geometry; and (c) $2^1A'$ and $3^1A'$ PESs, highlighting CI-6 at a bond angle close to that of the ground state minimum. All energies are defined relative to the ground state minimum energy geometry at E = 0.

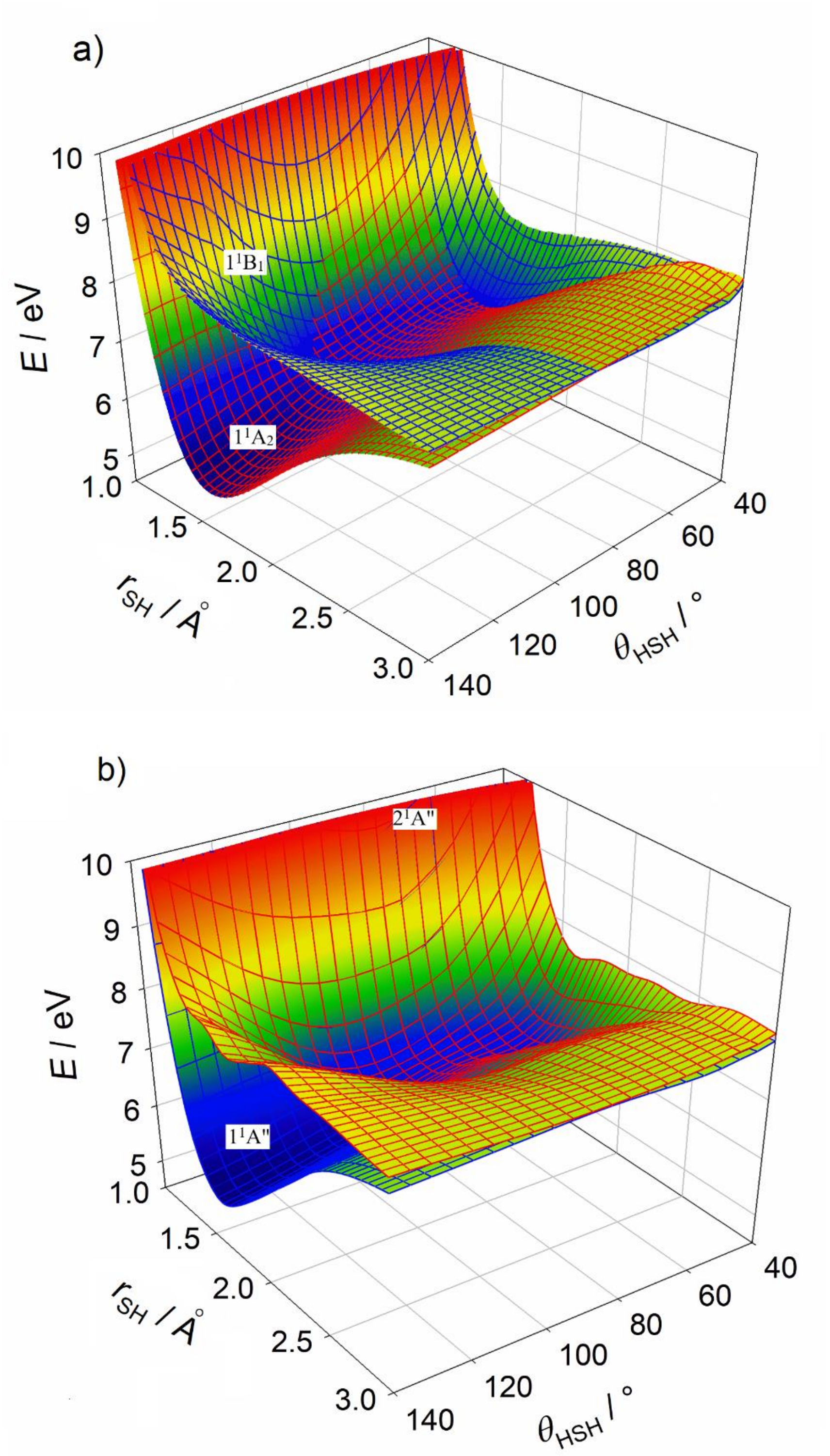

Fig. S13. The 3-D plots showing the topography of (a) the 1^1A_2 and 1^1B_1 PESs and (b) the adiabatic $1^1A''$ and $2^1A''$ PESs of H_2S in C_{2v} symmetry, plotted as functions of r_{SH} and θ_{HSH}.

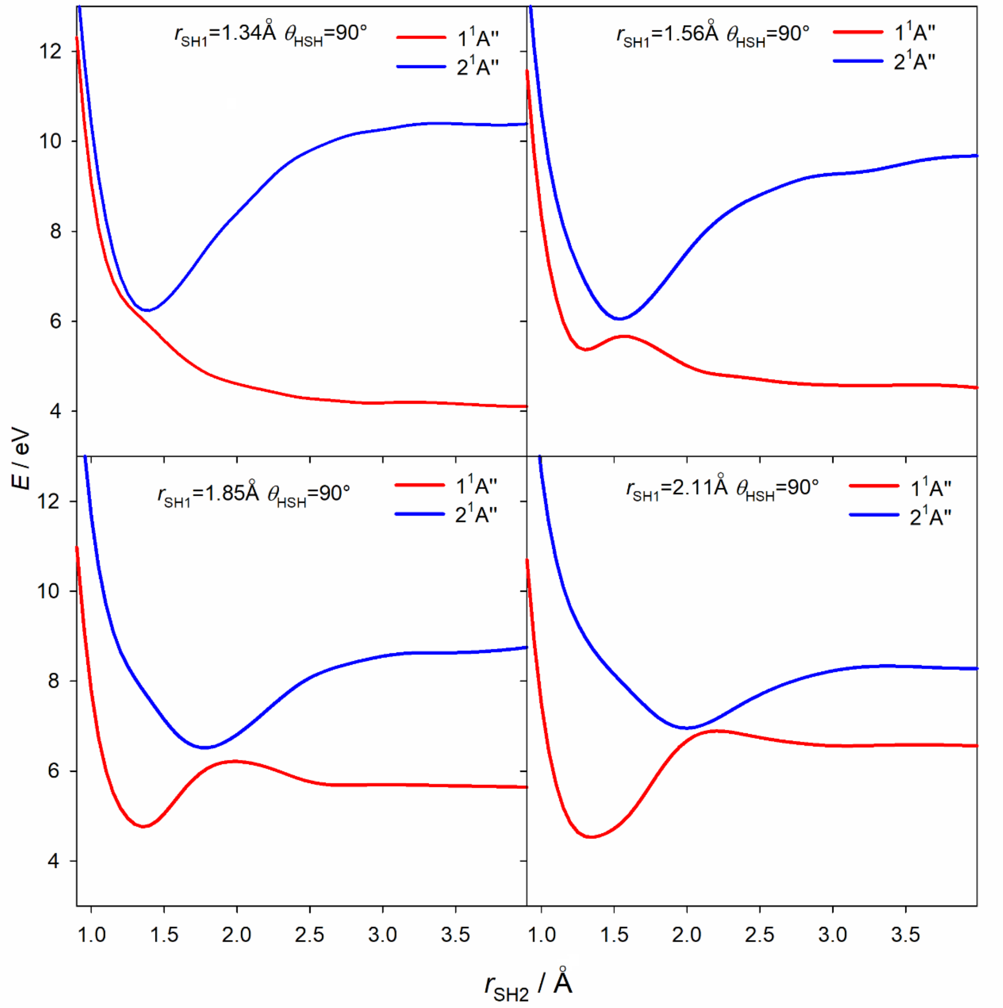

Fig. S14. The Cuts through the calculated PESs for the $1^1A''$ and $2^1A''$ states of H_2S, plotted as functions of r_{SH2}, with θ fixed at 90 °and r_{SH1} values of 1.34, 1.56, 1.85 and 2.11 Å.

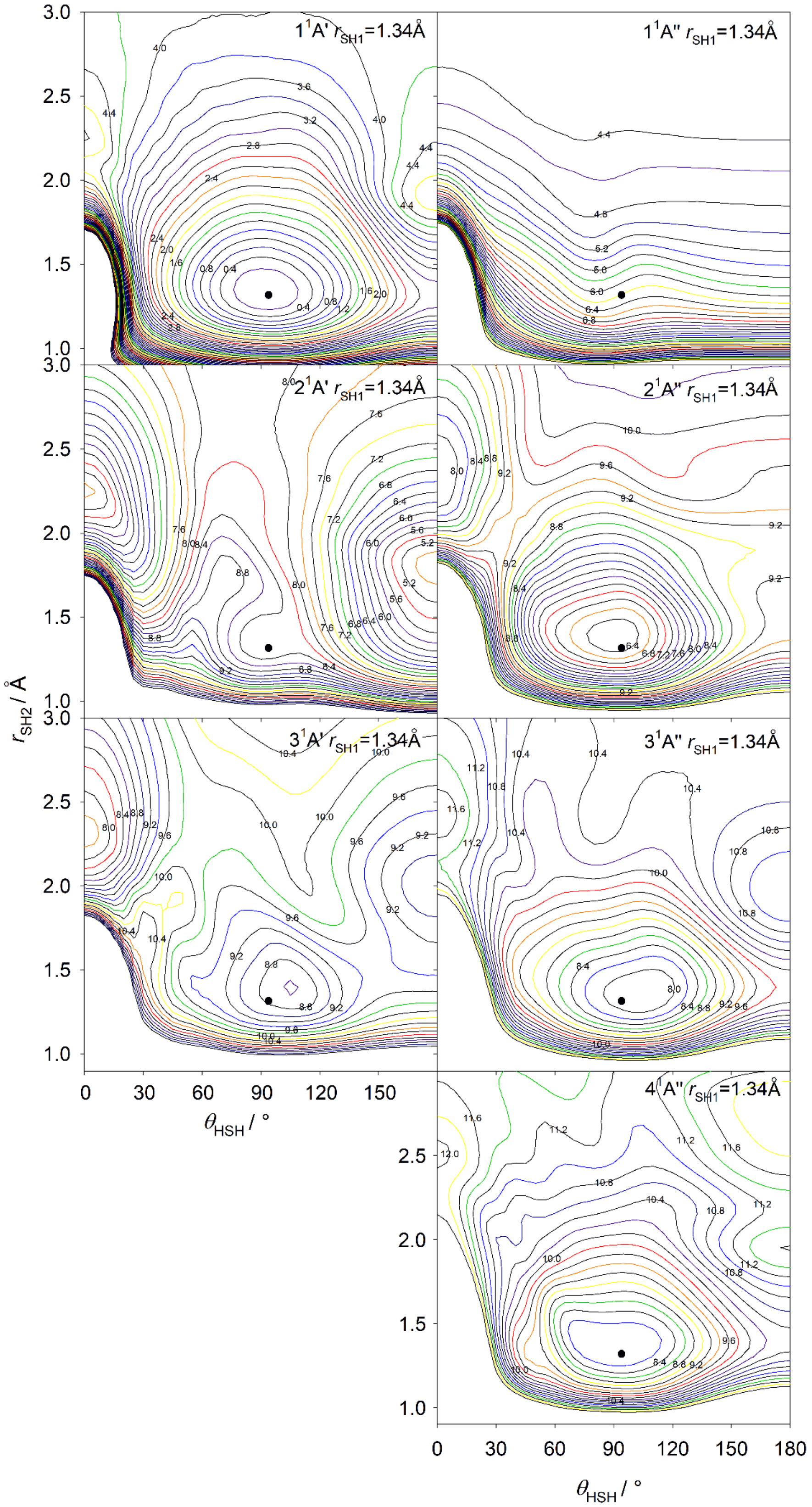

Fig. S15. The contour plots for the $1^1A'$, $2^1A'$, $3^1A'$, $1^1A''$, $2^1A''$, $3^1A''$ and $4^1A''$ PESs, plotted as functions of r_{SH2} and θ, with r_{SH1} fixed at the ground state equilibrium value (1.34 Å). The energy contours are labelled in eV, defined relative to the ground state minimum, the geometry of which is indicated by the black spot in each plot.

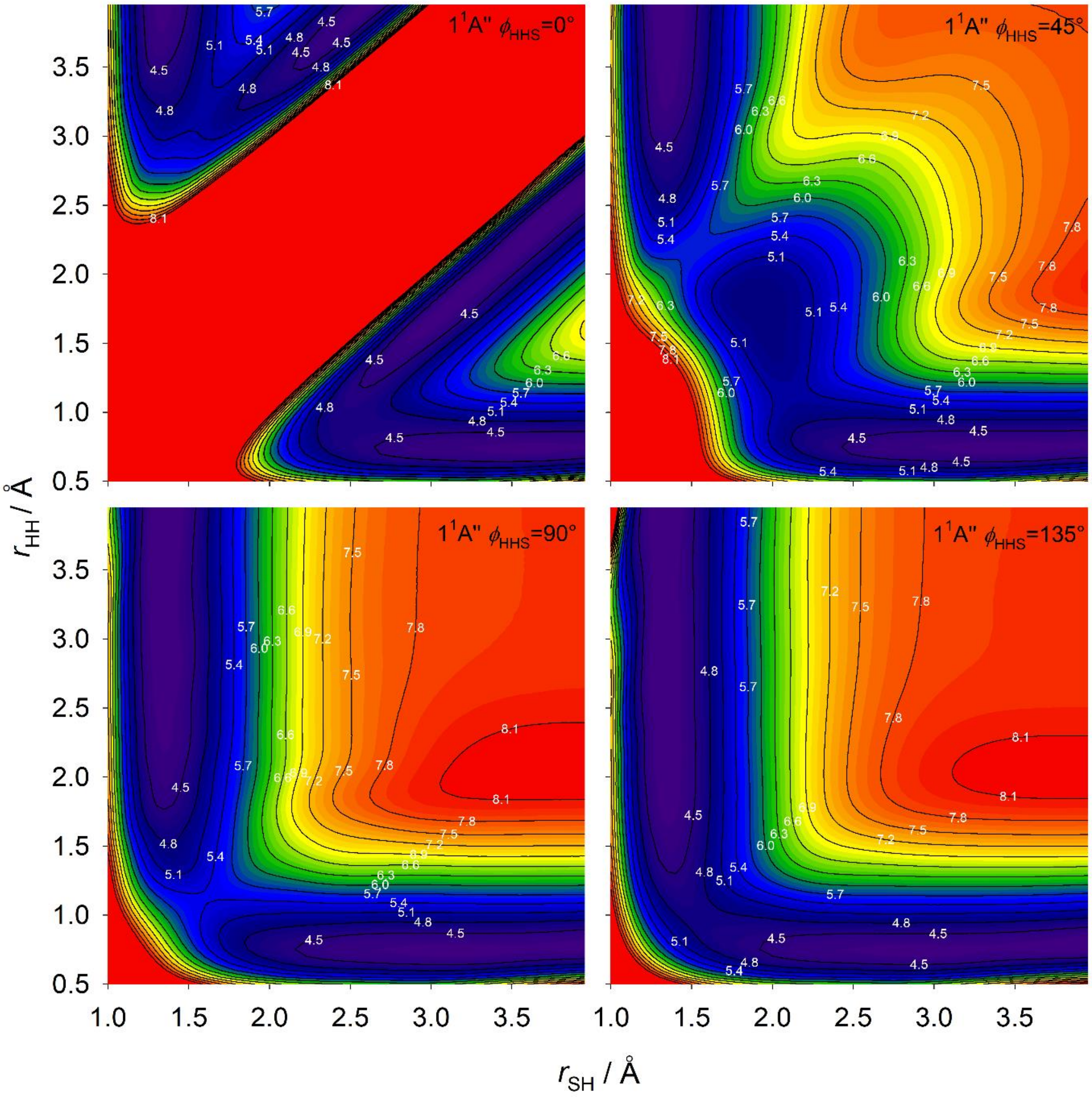

Fig. S16. The false colour plots showing the potential energy as functions of the H–H distance (r_{HH}) and the shorter S–H separation (r_{SH}) for ∠SHH bond angles ϕ = 0, 45, 90, and 135° for the $1^1A''$ PES. The energy contours are labelled in eV, defined relative to the ground state minimum.

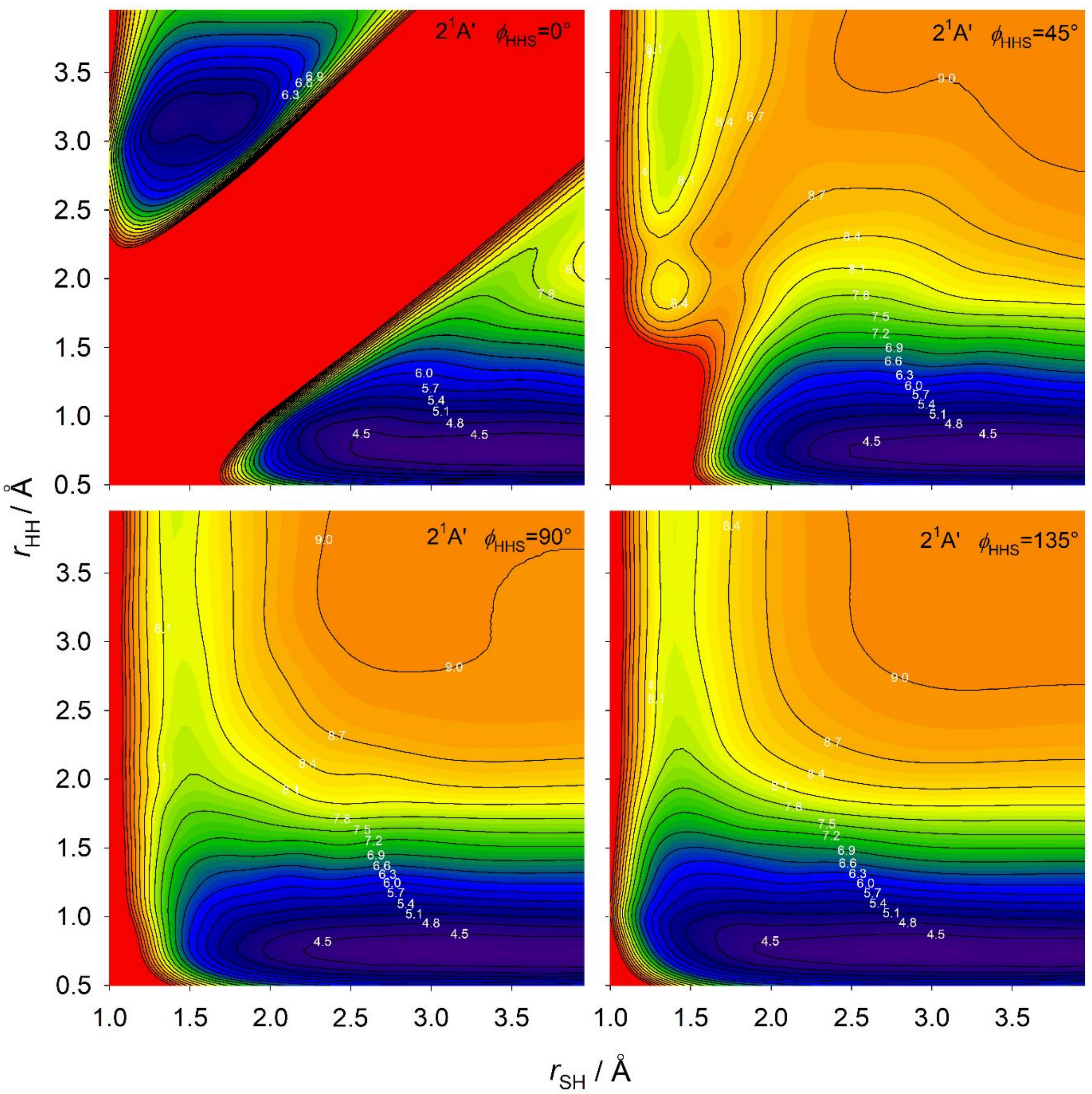

Fig. S17. The false colour plots showing the potential energy as functions of the H–H distance (r_{HH}) and the shorter S–H separation (r_{SH}) for ∠SHH bond angles $\phi = 0$, 45, 90, and 135° for the $2^1A'$ PES. The energy contours are labelled in eV, defined relative to the ground state minimum.

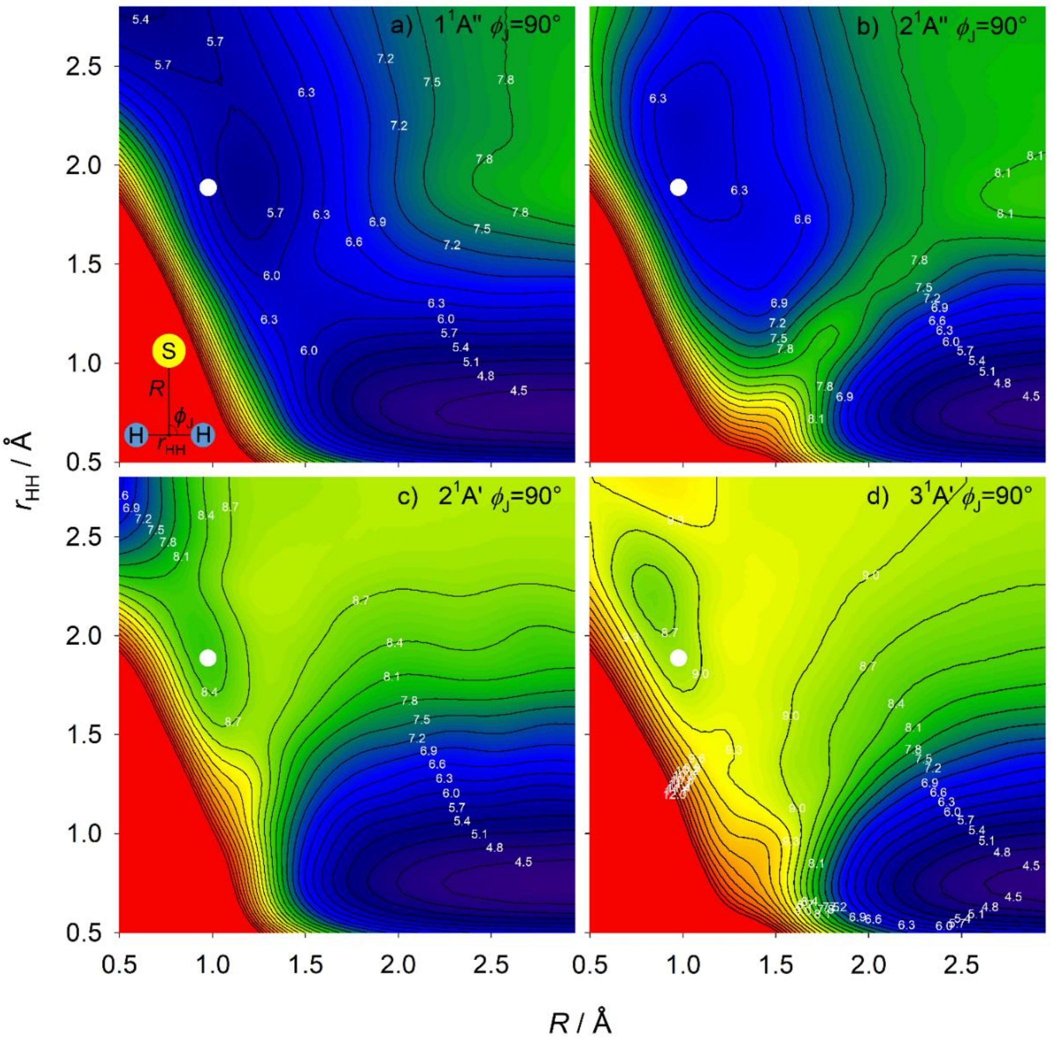

Fig. S18. The false colour plots showing the calculated (a) $1^1A''$, (b) $2^1A''$, (c) $2^1A'$ and (d) $3^1A'$ PESs plotted using Jacobi coordinates R (defining the distance between the S atom and the H_2 centre of mass) and the H–H bond length (r_{HH}) for an S–H_2 Jacobi angle of 90°. The vertical region is indicated by the white dot in each panel and the energy contours are labelled in eV, defined relative to the ground state minimum energy geometry at $E = 0$.

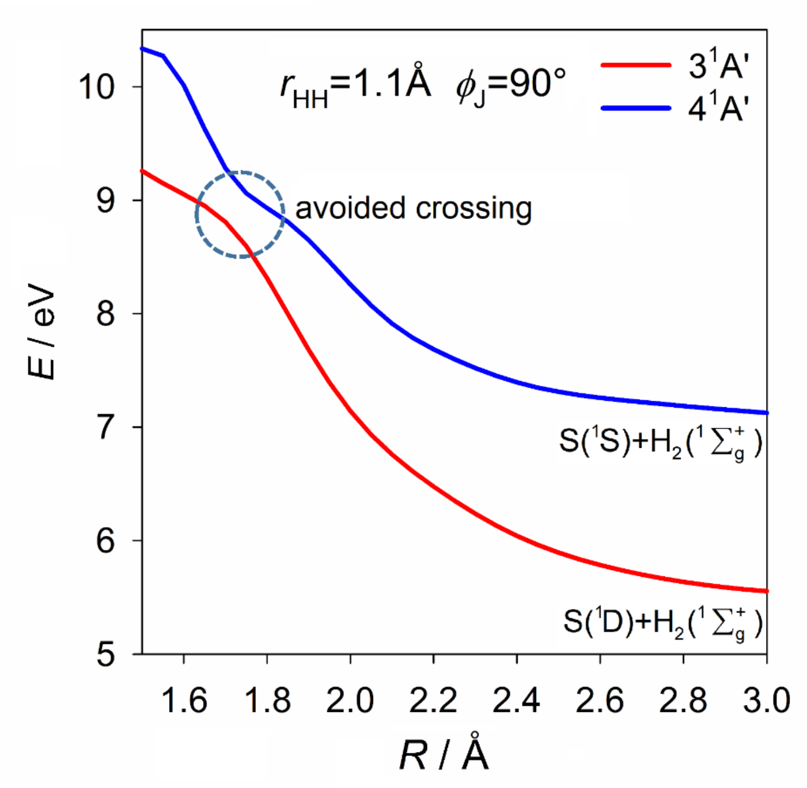

Fig. S19. The potential energy curves (PECs) for the $3^1A'$ and $4^1A'$ states as functions of the S + H_2 Jacobi coordinate R, with ϕ_J = 90° and r_{HH} = 1.1Å (a representative average bond length for $H_2(v'' = 5)$ products), with the avoided crossing between the two PECs at E ~9 eV circled. The energies are defined relative to the ground state minimum.

Reference

1 H. L. Wang, Y. Yu, Y. Chang, S. Su, S. R. Yu, Q. M. Li, K. Tao, H. L. Ding, J. Y. Yang, G. L. Wang, L. Che, Z. G. He, Z. C. Chen, X. A. Wang, W. Q. Zhang, D. X. Dai, G. R. Wu, K. J. Yuan and X. M. Yang, *J. Chem. Phys.*, 2018, **148**, 124301.

2 Y. Chang, S. Yu, Q. Li, Y. Yu, H. Wang, S. Su, Z. Chen, L. Che, X. Wang, W. Zhang, D. Dai, G. Wu, K. Yuan and X. Yang, *Rev. Sci. Instrum.*, 2018, **89**, 063113.

3 J. M. Zhou, Z. J. Luo, J. Y. Yang, Y. Chang, Z. G. Zhang, Y. Yu, Q. M. Li, G. K. Cheng, Z. C. Chen, Z. G. He, L. Che, S. R. Yu, G. R. Wu, K. J. Yuan and X. M. Yang, *Phys. Chem. Chem. Phys.*, 2020, **22**, 6260-6265.

4 Z. X. Li, M. Zhao, T. Xie, Z. J. Luo, Y. Chang, G. K. Cheng, J. Y. Yang, Z. C. Chen, W. Q. Zhang, G. R. Wu, X. G. Wang, K. J. Yuan and X. M. Yang, *J. Phys. Chem. Lett.*, 2021, **12**, 844-849.
